# HIVGenoPipe: a nextflow pipeline for the detection of HIV-1 drug resistance using a real-time sample-specific reference sequence

**DOI:** 10.1186/s12859-025-06201-5

**Published:** 2025-07-07

**Authors:** Thoai Dotrang, Brad T. Sherman, Lisheng Dai, Muhammad Ayub Khan, Helene C. Highbarger, Whitney Bruchey, Sylvain Laverdure, Michael W. Baseler, Tomozumi Imamichi, Robin L. Dewar, Weizhong Chang

**Affiliations:** 1 Laboratory of Human Retrovirology and Immunoinformatics, Frederick National Laboratory for Cancer Research, Frederick, MD 21702 USA; 2 Virus Isolation and Serology Laboratory, Frederick National Laboratory for Cancer Research, Frederick, MD 21702 USA; 3 Applied and Developmental Research Directorate, Frederick National Laboratory for Cancer Research, Frederick, MD 21702 USA

**Keywords:** Drug resistance, HIV-1, Mutation detection, Nextflow pipeline, Next generation sequencing, Sample-specific reference sequence

## Abstract

**Background:**

The emergence of HIV drug resistance is a challenge in controlling the acquired immunodeficiency syndrome (AIDS) pandemic caused by human immunodeficiency virus-1 (HIV-1) infection. Detection of drug resistance variants at minor frequencies can help to formulate successful antiretroviral therapy (ART) regimens for people living with HIV (PLWH) and reduce the emergence of drug resistance. Therefore, a pipeline which can accurately produce consensus nucleotide sequences and identify drug resistance mutations (DRMs) at defined frequency thresholds will be helpful in the treatment of PLWH, analysis of virus evolution, and the control of the pandemic.

**Results:**

We have developed a pipeline, HIVGenoPipe, to determine HIV drug resistance variants within the *gag-pol* region above user-defined frequencies for HIV-1 samples sequenced using Illumina technology. The pipeline has been validated by comparing its results with the results generated by a widely used pipeline, HyDRA, which is limited to the *pol* region, and with the results generated by Sanger sequencing technology using the same set of 30 samples. The variant frequency used to generate ambiguous consensus sequences in HIVGenoPipe is more accurate than other pipelines because a sample-specific reference, which is generated in real-time with a novel hybrid strategy of de novo and reference-based assembly, is used for the frequency calculation, leading to more accurate drug resistance calls for use by clinicians. In addition, since Nextflow is used as the pipeline platform, HIVGenoPipe inherently has great portability, scalability and reproducibility; and the components can be updated or replaced independently if required.

**Conclusions:**

We developed HIVGenoPipe for the detection of HIV-1 drug resistance. It constructs more accurate gag-pol consensus sequences, leading to improved detection of DRMs. HIVGenoPipe is open source and freely available under the MIT license at https://github.com/LHRI-Bioinformatics/HIVGenoPipe. The current release (v1.0.1) is archived and available at https://doi.org/10.5281/zenodo.15528502.

**Supplementary Information:**

The online version contains supplementary material available at 10.1186/s12859-025-06201-5.

## Background

Human immunodeficiency virus-1 (HIV-1) is the virus causing acquired immunodeficiency syndrome (AIDS) [1], a pandemic having claimed more than 42 million human lives over the past four decades based on World Health Organization (WHO) data [2]. There has been tremendous progress in the treatment and control of HIV infection. Several types of drugs have been developed including those targeting different viral proteins such as nucleoside reverse transcriptase inhibitors (NRTIs) and non-nucleoside reverse transcriptase inhibitors (NNRTIs) targeting reverse transcriptase (RT) [3], protease inhibitors (PIs) targeting protease (PR) [4, 5], integrase strand transfer inhibitors (INSTIs) targeting integrase (IN) [6]; and drugs targeting host-virus interaction such as fusion inhibitors, CCR5 antagonists [7] and post-attachment inhibitors. In 2022, Lenacapavir was approved to treat people living with HIV (PLWH) [8, 9]. It can bind directly to the interface between capsid subunits, leading to the inhibition of HIV-1 capsid function and HIV-1 replication. These drugs make it possible to prescribe an effective antiretroviral therapy (ART) regimen for PLWH [10–12]. However, the pandemic is still ongoing with an estimated 630,000 deaths from HIV-related causes and an estimated 1.3 million new HIV infections in 2023 [2]. One reason for this is the high mutation rate of HIV due to the lack of a proof-reading function of the HIV RT and the high propagation rate of the virus, leading to the quick selection of drug resistance mutations (DRMs) after antiviral treatment [13, 14]. To overcome this problem, there is a need to develop new effective drugs [15] and improve the detection of drug resistance in order to formulate successful ART regimens for PLWH and eliminate unnecessary antiviral drug use, which can reduce the occurrence of drug resistance [16–18]. Sanger sequencing was the first available method to be used for HIV genotyping such as HIV Seq [19, 20] and VircoTYPE HIV-1 [21]. The development of next generation sequencing (NGS) around 2005 made it possible to genotype low abundance variants in HIV samples. Many HIV drug resistance determination workflows using NGS data [22] have been developed such as HyDRA [23], PASeq [24], QuasiFlow [25], HIV-DRIVES [26] and DEEPGEN HIV [27]. The Stanford HIV Drug Resistance Database (HIVdb) [28], which is commonly used for HIV-1 drug resistance interpretation, also offers a pipeline for analysis of NGS data. The critical step of these workflows is to assemble the HIV genome sequence which encodes PR, RT, RNase H (RH) and IN (usually just the *pol* gene). There are two different methods to assemble genome sequences: de novo assembly and reference-based assembly [29]. De novo assembly builds a genome sequence from scratch, without any prior knowledge or reference genome. This method pieces together small fragments of DNA (sequencing reads) by identifying overlapping sequences and assembling them into longer contiguous sequences, or contigs. It is particularly useful for organisms without a closely related reference genome, but it is computationally more challenging and can produce more fragmented assemblies. In contrast, reference-based assembly uses an existing, known reference genome to align and map sequencing reads. This approach is faster and less computationally intensive because it relies on the similarity between the sample genome and the reference, but it may introduce bias if the reference genome is significantly different from the sample. In this study, we have developed a pipeline, HIVGenoPipe, which uses a novel multiple-step hybrid assembly procedure to assemble the *gag-pol* region, limiting the bias introduced by reference-based assembly. The pipeline provides more accurate ambiguous base calls at user-defined frequency thresholds, leading to more accurate drug resistance interpretation. The accurate consensus sequences generated by this pipeline are valuable for the research of virus evolution. In addition, HIVGenoPipe can also detect the resistance to antiviral drugs targeting proteins encoded by the *gag* region sequence such as Lenacapavir, which targets capsid.

## Methods

### Overview of HIVGenoPipe

**Fig. 1:**
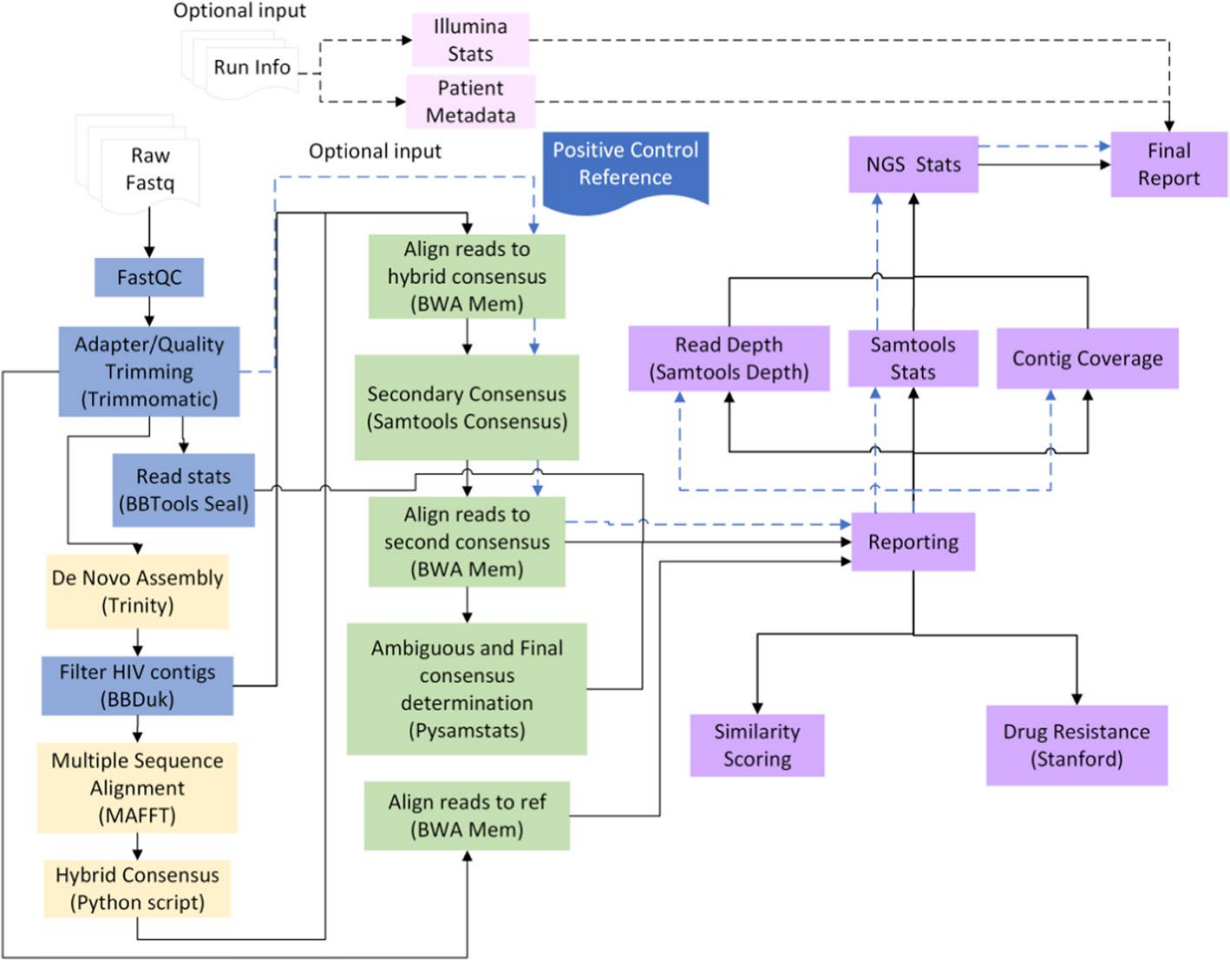
HIV Genotyping Pipeline (HIVGenoPipe) Workflow. The solid arrows show the main workflow from the raw data to final reports. The blue dashed arrows indicate the optional subworkflow to provide QC statistics with positive control. The black dashed arrows indicate optional Illumina sequencing run statistics and/or patient metadata reporting subworkflow. Blue processes indicate read trimming/filtering steps. Yellow processes indicate *de novo*assembly steps leading into the hybrid consensus creation. Green processes highlight the subsequent alignment steps and consensus determination. Purple processes represent reporting or statistics collection steps

In this study, we developed the HIVGenoPipe pipeline to accurately assemble HIV genomic consensus sequences at user-defined variant frequencies (default: 5% and 15%) and characterize their drug resistance profiles (Fig. 1). The HIVGenoPipe pipeline was developed using the Nextflow framework [30]. The pipeline begins with several pre-processing steps typically performed on Illumina (MiSeq) paired-end short reads. First, FastQC (version 0.12.1) [31] together with MultiQC (v1.12) [32] are used to check sequencing data quality. Trimmomatic (v0.39) [32] is then used to perform quality trimming and filtering. The processed sequencing reads (cleaned reads) are then used in de novo assembly to form contigs with Trinity (v.2.13.2) [33]. A de novo method is used in this first assembly in order to avoid bias that occurs with reference-based alignment. At the same time, Sequence Expression AnaLyzer (SEAL) from the BBTools suite (v39.13) [34] is used for gathering preliminary read statistics. De novo assembly typically does not form full-length contigs, especially in the presence of quasispecies. Therefore, we constructed a hybrid HIV consensus sequence using the following steps: The contigs from Trinity are filtered to remove any non-HIV contigs using BBDuk [34]. The remaining contigs are then aligned to the HIV-1 reference (HIV-1 strain HXB2 *gag-pol* region, NCBI Accession No: K03455.1 737-5219) using MAFFT (v7.508), a multiple sequence alignment tool [35]. A hybrid consensus sequence is generated by flattening the aligned contigs and filling in any gaps between contigs with the reference sequence using an in-house python script that employs Biopython (v1.71) [36]. This hybrid consensus is then used as the reference sequence to align the cleaned reads using BWA-MEM (v0.7.17) [37] and the alignment is used to generate an intermediate consensus sequence with SAMtools consensus (v1.15.1) [38]. To obtain accurate consensus sequences based on nucleotide frequency at each position, the cleaned reads are again aligned to the intermediate SAMtools consensus using BWA-MEM with a mismatch penalty set to 3 (default = 4). Next, pysamstats (v1.1.2) [39] is used to determine the read statistics for each position based on this sequence alignment. And finally, an in-house script is used to make base calls based on the pysamstats statistics, including ambiguous bases, at the user-defined variant frequency thresholds (ambiguity threshold) and produce consensus sequences at these ambiguity levels along with a report indicating positions flagged as insertions or deletions (indels). The minimum read support threshold at this step is set to 10 for each variant nucleotide at each position, e.g. 200 × for 5% and 67 × for 15%. The percentage above the threshold at a given position within the consensus sequence is calculated and reported in the depth QC table. A percentage < 90 will lead to the labeling of “fail” at the end of consensus file name to warn the user. The sequences are then submitted to the Stanford HIVdb [40] through the Sierra Web Service 2 (v1.1) [42] to determine possible drug resistance. Depth plots, drug resistance reports, and other summary statistics are generated at the end of the workflow.

A user may optionally include sample metadata as well as Illumina run statistics as input to have them in the final report. The positive control subworkflow is another option that will align any positive control samples to an additional control reference sequence, separate from the reference used for consensus determination. By default, HXB2 *gag-pol* region is used as a reference for consensus determination while NL4-3 is used as a positive control reference because we used an NL4-3 virus sample (NL4-3 is lab synthetic strain) as a positive control. This subworkflow was designed to analyze positive control samples for quality assurance of the sequencing run as they are used alongside clinical samples.

### Ethics statement

All experimental procedures in these studies were approved by the National Cancer Institute at Frederick and the National Institute of Allergy and Infectious Diseases (NIAID) Institutional Review Boards and performed in accordance with the relevant guidelines and regulations. Samples were obtained from 30 participants who were enrolled in following institutional review board-approved NIH studies: Clinical and Immunologic Monitoring of Patients With Known or Suspected HIV Infection (ClinicalTrials registration number NCT00789009), A Study to Evaluate the Safety and Effects of Repeated Doses of 3BNC117-LS and 10–1074-LS on Persistent Viral Reservoirs in People Living With HIV and on Suppressive Antiretroviral Therapy (ClinicalTrials registration number NCT05612178), A Trial of Anti-CD4 Antibody UB-421 in Combination With Optimized Background Antiretroviral Therapy in Patients With Multi-Drug Resistant HIV-1 Infection (ClinicalTrials registration number NCT05582694) and Tissue Biopsy and Imaging Studies in HIV-Infected Patients (ClinicalTrials registration number NCT00001471). The participant provided written informed consent.

### Generation of the test dataset

A total of 30 plasma samples were obtained from PLWH enrolled in NIAID clinical trials and undergoing ART, and written informed consent for study procedures and sample analysis was provided. Several samples exhibited multidrug resistance, including resistance to NRTIs, NNRTIs, PIs, and INIs. Approximately half of the samples had viral loads in plasma ranging from 1000 to 5000 copies/mL, while the remaining samples had viral loads in plasma exceeding 10,000 copies/mL (Table S1). Plasma samples from these individuals were used to generate MiSeq and Sanger sequencing data using standard procedures (Supplemental Method).

MiSeq sequencing data, ranging from 24,714 to 163,084 paired reads (Table S1), were used as input for HIVGenoPipe and published reference pipeline, HyDRA. The Sanger sequencing data produced from Applied Biosystems 3500 Dx Series Genetic Analyzers (Thermo Fisher Scientific, Waltham, MA, USA) was assembled into consensus sequences using Sequencher (Version.5.4.6, Gene Codes Corporation, Ann Arbor, MI, USA) with default options except the base confidence threshold, which was reduced to 15 from 25 at both ends. Due to the existence of quasispecies in the HIV-1 population, a full-length sequence could not be formed with this default set of options for some samples. To overcome this, we relaxed the threshold further by increasing the ambiguous base number to 4 or 5 from 3 within 25 bases at both ends. Then, we replaced the overlapping region of the full-length sequence with contigs of better quality. The modified full-length amplicon sequences were used as another reference sequence to validate the HIVGenoPipe results.

Raw MiSeq sequencing data have been submitted to NCBI SRA under accession number: BioProject PRJNA1218827. The Sanger sequencing assemblies are provided in FASTA format in Supplemental Data 1.

To avoid confusion, the coordinates of all positions of the HIV sequences in this work were annotated based on the full-length HIV-1 HXB2 sequence (NCBI Accession: K03455.1).

### Comparison of the test results from HIVGenoPipe and HyDRA pipeline

To validate HIVGenoPipe, we compared the results of this pipeline with the widely used HyDRA pipeline. MiSeq sequencing data from the 30 plasma samples described above were submitted to the HyDRA web application with comparable run settings and the resulting consensus sequences and alignment files were downloaded. The HyDRA sequencing reads were extracted from the downloaded alignment files in the HyDRA output. The HyDRA sequencing reads were aligned to the output consensus sequences from both pipelines with BWA or Bowtie2 (v2.5.4) [41], to judicate the discrepancies in drug resistance reports and consensus sequences from both pipelines.

To ensure that the drug resistance interpretation was performed with the same version of the Stanford drug resistance algorithm (V9.8), the consensus sequences from both pipelines were sent to the Stanford Sierra Web Service 2 and the output, in JSON format, was compared programmatically.

The consensus sequences from both pipelines at three variant frequencies (2%, 5% and 15%) were compared with BLAST [42]. Discrepancies were judicated by assessing final alignment files from the two pipelines and several alignment files generated for comparison: HIVGenoPipe filtered and trimmed reads aligned to the SAMtools consensus by BWA-MEM, HyDRA filtered reads aligned to HXB2 *pol* by Bowtie2 (HyDRA final alignment file), HyDRA reads aligned to the output consensus sequence of HIVGenoPipe or HyDRA by BWA-MEM, HyDRA reads aligned to HXB2 *pol* by BWA-MEM, and HyDRA reads aligned to the HyDRA output consensus by Bowtie2. Nucleotide base positions of interest were visualized with IGV [43].

### Comparison of consensus sequences from HIVGenoPipe with the assembly sequences from sanger sequencing data

Sanger sequencing has been accepted as the validation standard for NGS DNA sequencing and we used it as another validation for HIVGenoPipe. Additionally, HyDRA only analyzes ~ 2.8 kb of the *pol* region and our amplicon covers ~ 4.5 kb of the *gag-pol* region which can be fully validated by comparing with Sanger consensus sequences. The consensus sequences from Sanger sequencing data and the ambiguous consensus sequences acquired from HIVGenoPipe at a 15% ambiguity threshold were compared with BLAST as described above. Discrepancies were judicated using the alignment files visualized with IGV and sequence traces in Sanger sequencing chromatograms.

## Results

### Development of HIVGenoPipe and output of the pipeline

We developed HIVGenoPipe to determine the drug resistance variants in HIV-1 samples as described above (Fig. 1). Each module of HIVGenoPipe will return its output files into its respective directory and all intermediate files are also made available to the user (Figure S1). The final reporting files include three sets: (1) Final consensus sequences (majority consensus, ambiguous sequence at defined frequency thresholds); (2) Drug resistance reports in three separate reports (mutations, drug resistance, and protein sequences) for each sample at each defined ambiguity level as well as for the majority consensus; (3) Indel reports containing indel information for the relevant positions. Depth QC statistics are also included in this report. The important QC files for users include five sets: (1) Nextflow run reporting files; (2) MultiQC report for read quality; (3) Read depth plot for read depth at each position in alignment to the SAMtools consensus sequence; (4) Sample similarity matrix: heatmap matrix images to show sample to sample similarity; (5) Final report in ‘report’ folder, a comprehensive report detailing read statistics, alignment statistics, and the consensus sequences. If positive or negative control samples are included in the sequencing data, the statistics for these control samples are included in the final report (Table S3). The output files described above for the 30 test samples are provided as examples (Supplemental Data 2).

The pipeline can be run in any Unix or Linux system with Docker or Singularity. The pipeline has been tested to run with Docker and Singularity successfully. Our VM system settings: Oracle Linux Server V 8.10, RAM 196 G, 64 bits, 48 CPU. The analysis of 30 test samples plus one negative control and one positive control on this system takes under 30 min with Docker. Our server utilizes a network with a download speed of ~ 150 Mbps and upload speed of ~ 600 Mbps.

### Result of HIVGenoPipe comparison to HyDRA

To validate HIVGenoPipe, we compared results from the well-accepted HIV drug resistance pipeline HyDRA using the same MiSeq sequencing data generated from the 30 plasma samples as described above. The HyDRA analysis focuses on a ~ 2.8 kb portion of the *pol* region within the HIV-1 genome, while HIVGenoPipe was developed using a ~ 4.5 Kb amplicon of the *gag-pol* region. Therefore, the comparison only validated the ~ 2.8 *pol* region in which they overlap. We compared the results from the two pipelines with two default frequency thresholds of HIVGenoPipe: 5% and 15%.

**Fig. 2:**
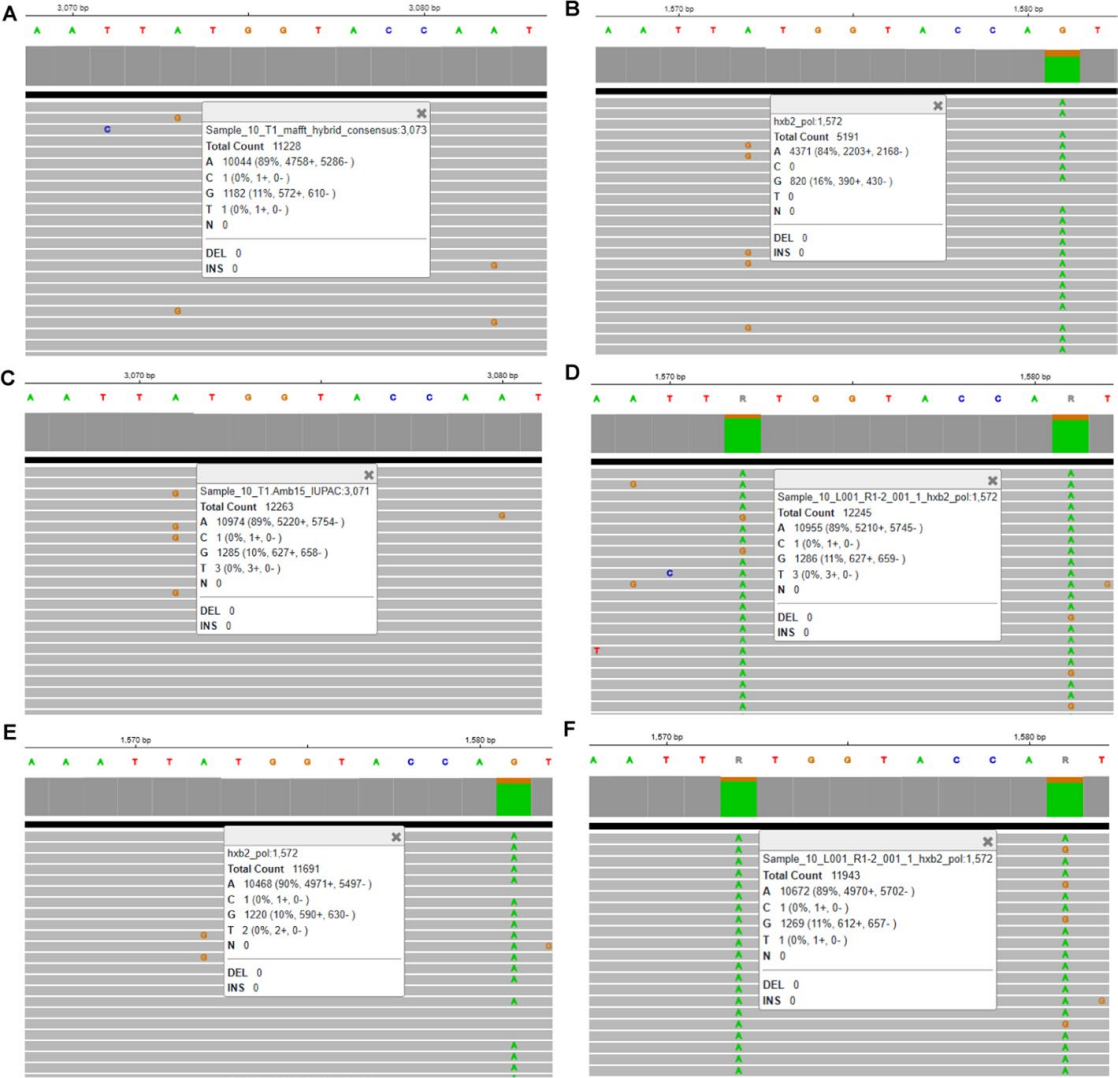
Sample-specific reference in HIVGenoPipe results in accurate ambiguous base call: Sample 10 at HXB2 position 3824 (corresponding to coordinates on full length HXB2). **A** Final alignment in HIVGenoPipe. Sequencing reads aligned to the intermediate Samtools consensus with BWA (mm *p* = 3). **B** Final HyDRA alignment. HyDRA reads aligned to HXB2 with Bowtie2. **C** HyDRA reads aligned to the HIVGenoPipe final consensus (15% ambiguity threshold) with BWA (mm *p* = 3). **D** HyDRA reads aligned to the HyDRA final consensus (15% ambiguity threshold) with BWA (mm *p* = 3). **E** HyDRA reads aligned to HXB2 with BWA (mm *p* = 3). **F** HyDRA reads aligned to the HyDRA final consensus (15% ambiguity threshold) with Bowtie2

**Fig. 3:**
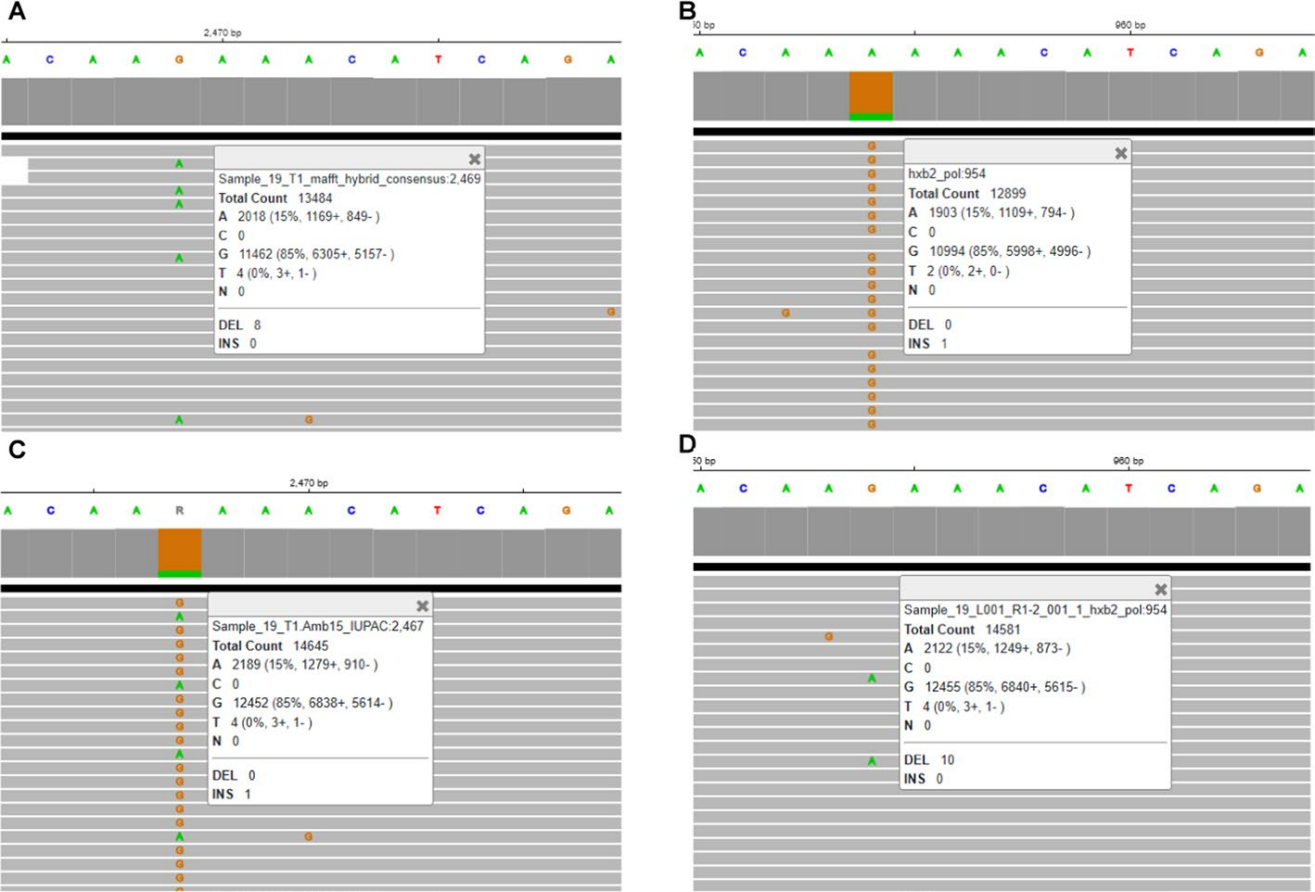
The position with different base call with minor variant frequency at the borderline: Sample 9 at HXB2 position 3206 (corresponding to coordinates on full length HXB2). **A** Final alignment in HIVGenoPipe. Sequencing reads aligned to the intermediate Samtools consensus with BWA (mm *p* = 3). **B** Final HyDRA alignment. HyDRA reads aligned to HXB2 with Bowtie2. **C** HyDRA reads aligned to the HIVGenoPipe final consensus (15% ambiguity threshold) with BWA (mm *p* = 3). **D** HyDRA reads aligned to the HyDRA final consensus (15% ambiguity threshold) with BWA (mm *p* = 3)

**Fig. 4:**
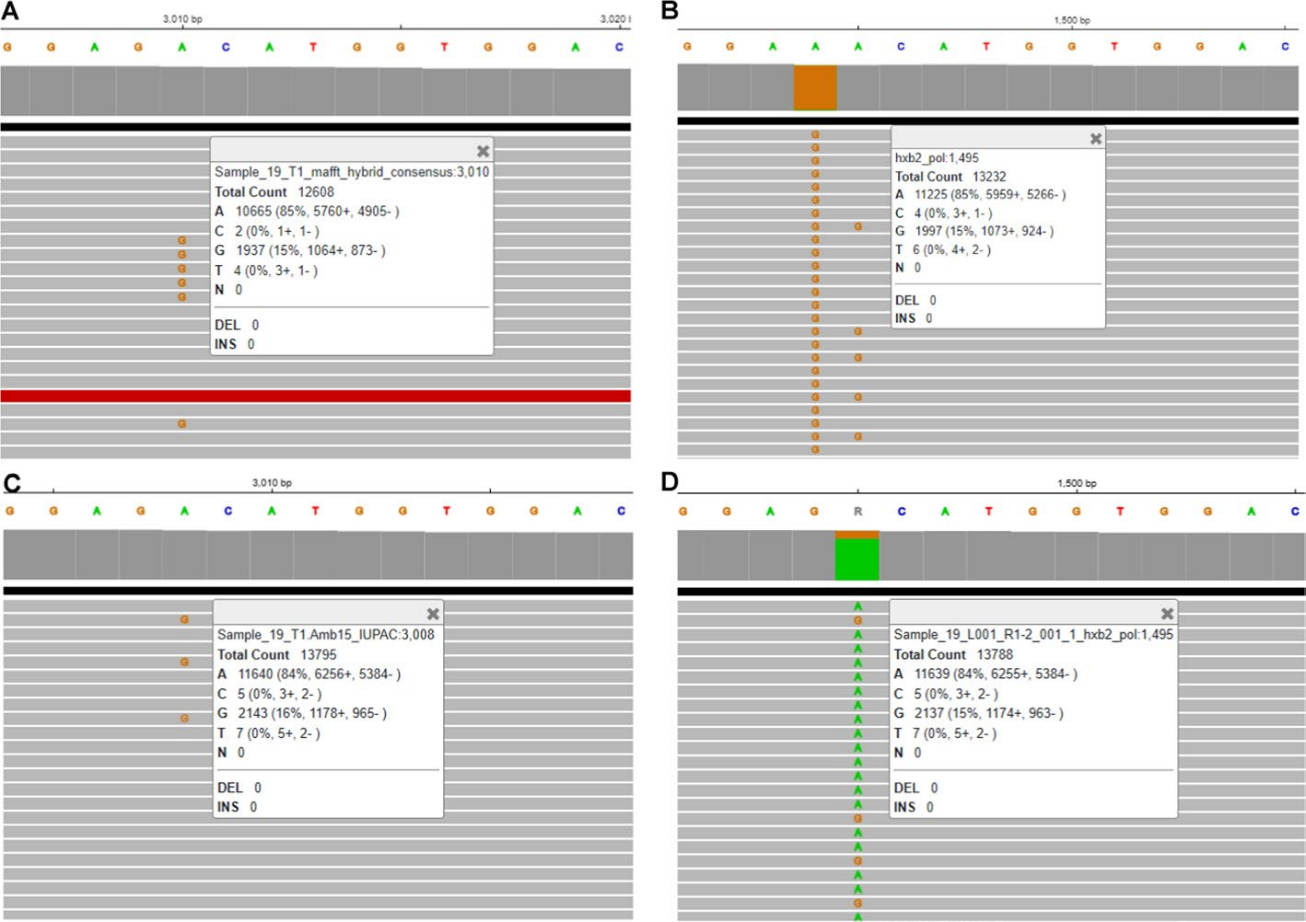
The position of different base call with minor variant frequency at the borderline: Sample 19 at HXB2 position 3747 (corresponding to coordinates on full length HXB2). **A** Final alignment in HIVGenoPipe. Sequencing reads aligned to the intermediate Samtools consensus with BWA (mm *p* = 3). **B** Final HyDRA alignment. HyDRA reads aligned to HXB2 with Bowtie2. **C** HyDRA reads aligned to the HIVGenoPipe final consensus (15% ambiguity threshold) with BWA (mm *p* = 3). **D** HyDRA reads aligned to the HyDRA final consensus (15% ambiguity threshold) with BWA (mm *p* = 3)

**Fig. 5:**
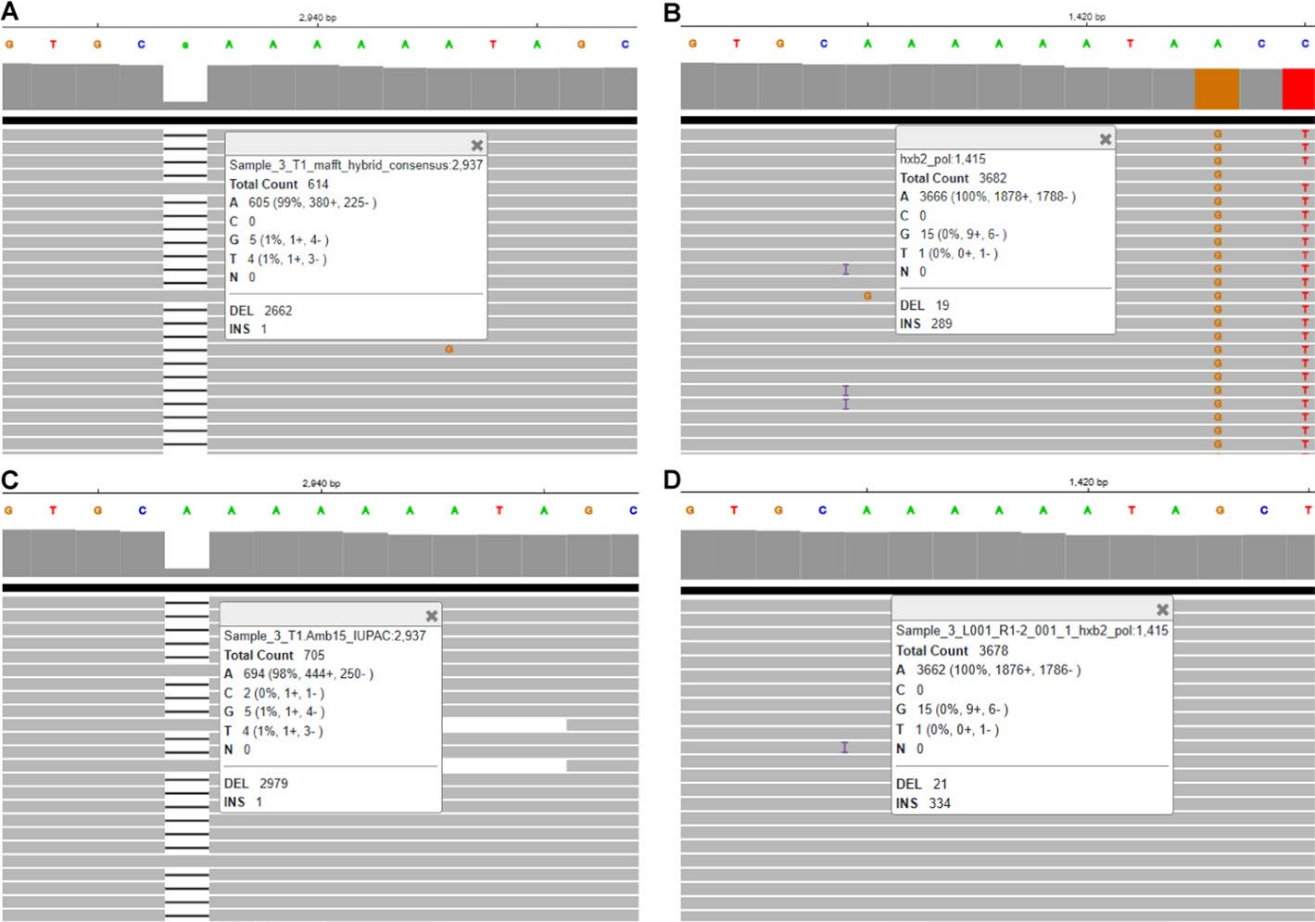
The position of different base call caused by the inclusion of insertion with minor abundancy in HIVGenoPipe: Sample 3 at HXB2 position 3667 (corresponding to coordinates on full length HXB2). **A** Final alignment in HIVGenoPipe. Sequencing reads aligned to the intermediate Samtools consensus with BWA (mm *p* = 3). **B** Final HyDRA alignment. HyDRA reads aligned to HXB2 with Bowtie2. **C** HyDRA reads aligned to the HIVGenoPipe final consensus (15% ambiguity threshold) with BWA (mm *p* = 3). **D** HyDRA reads aligned to the HyDRA final consensus (15% ambiguity threshold) with BWA (mm p = 3)

**Fig. 6:**
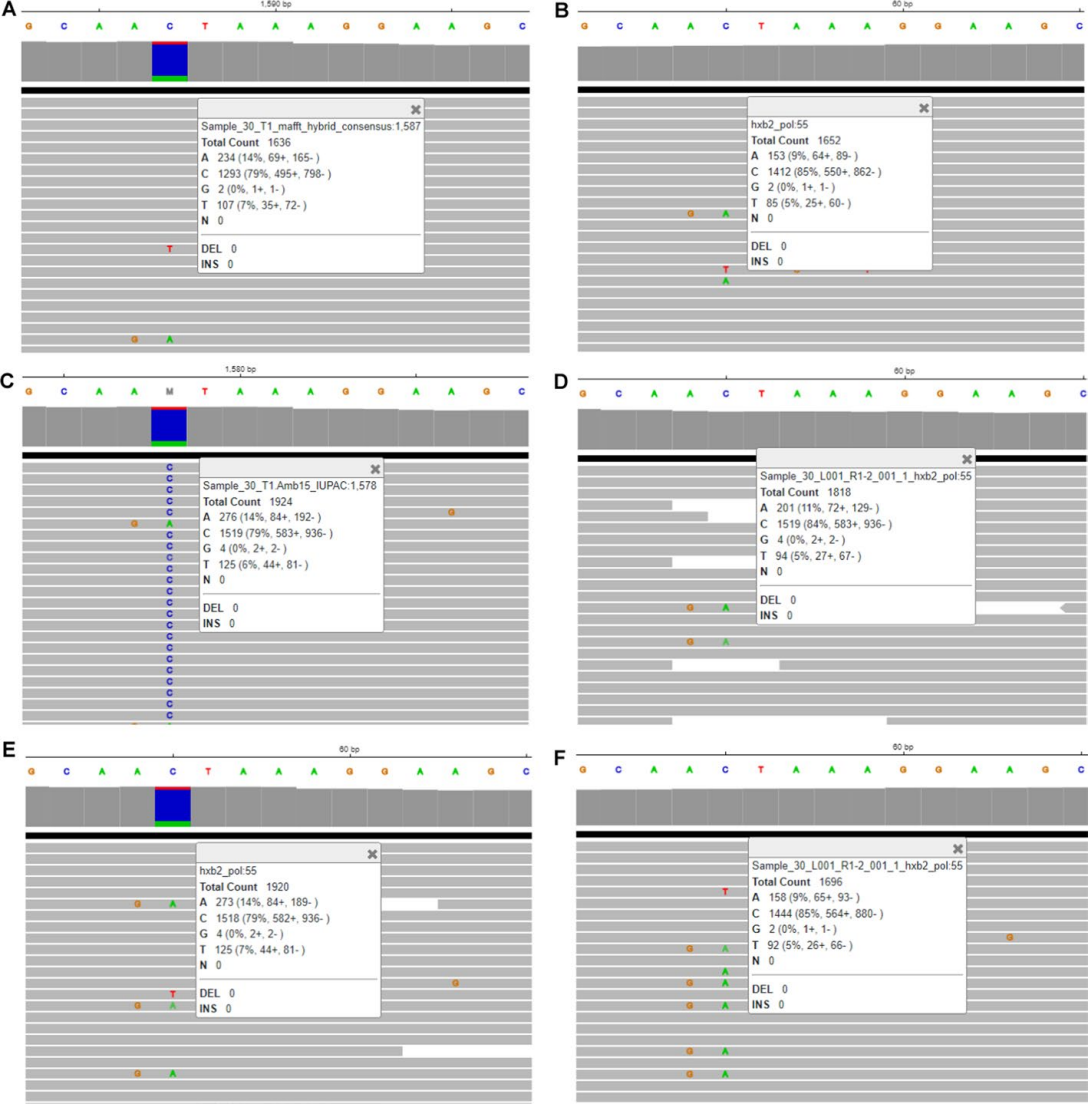
The position of different base call in HIVGenoPipe and HyDRA: Sample 30 at HXB2 position 2307 (corresponding to coordinates on full length HXB2). **A** Final alignment in HIVGenoPipe. Sequencing reads aligned to the intermediate Samtools consensus with BWA (mm *p* = 3). **B** Final HyDRA alignment. HyDRA reads aligned to HXB2 with Bowtie2. **C** HyDRA reads aligned to the HIVGenoPipe final consensus (15% ambiguity threshold) with BWA (mm *p* = 3). **D** HyDRA reads aligned to the HyDRA final consensus (15% ambiguity threshold) with BWA (mm *p* = 3). **E** HyDRA reads aligned to HXB2 with BWA (mm *p* = 3), **F** HyDRA reads aligned to the HyDRA final consensus (15% ambiguity threshold) with Bowtie2

When comparing the consensus sequences generated by HIVGenoPipe and HyDRA at a frequency of 15% for the 30 samples using BLAST, 65 mismatches were observed (Supplemental Data 3). Only one of these, in Sample 20 (HXB2 position 590), leads to a drug resistance variant, NNRTI ~ RT ~ A98AG, which was characterized by HIVGenoPipe, but not HyDRA. These 65 mismatches can be classified into four types. The first type arises from the differences in the reference sequences used during alignment for final consensus calling. HIVGenoPipe utilizes an intermediate SAMtools consensus sequence that closely resembles the sample's actual sequence, while HyDRA relies on the HXB2 genome as a reference. This difference accounts for 35 of the 65 observed mismatches (Supplemental Data 3). A sample 10 mismatch at HXB2 position 3824, provides a good example of the impact that the reference can have on the frequency and thus the final consensus (Fig. 2). For this position, we observe that the HIVGenoPipe BWA-MEM alignment, using the SAMtools intermediate consensus as a reference, maps 11,228 reads with a minor variant G at a frequency of 11% (1,182 reads) with the base called as A (Fig. 2A) while the HyDRA Bowtie2 alignment, using HXB2 as the reference, maps 5,191 reads at the position with a minor variant G at a frequency of 16% (820 reads) with the base called as R (Fig. 2B). The non-sample-specific reference (HXB2) had less reads aligned to the region than the sample-specific reference. Interestingly, we observed that the alignment of HyDRA reads to the final consensus sequences from both pipelines using BWA-MEM showed very similar variant frequencies and mapped read counts to each other and to the HIVGenoPipe alignment (Fig. 2C and D). This would lead to all having the same base call of A, at the 15% threshold, demonstrating that HIVGenoPipe made the correct base call at this position. Additionally, we found that at this position, a similar frequency can be obtained using BWA-MEM, even when using HXB2 as the reference (Fig. 2E). Finally, when we used the final HyDRA consensus sequence as the reference, both BWA-MEM and Bowtie2 also produced similar frequencies (Fig. 2D and F), highlighting the importance of using sample-specific reference sequences to generate the best possible consensus at the defined frequency levels. The second type of mismatch involves cases where the minor variant frequency hovers near the ambiguity threshold, where small differences in filtered mapping reads between the two pipelines caused slight shifts in the minor variant frequency, pushing it just above or below the threshold (Figs. 3 and 4). This contributed to 31 out of the 65 mismatches. We also observed that three mismatches were caused by both the reference sequences used in the final alignment and the frequencies being at the borderline: Sample 17—pos 3423, Sample 17—pos 3935 and Sample 19—pos 3839 (Supplemental Data 3). The third type of discrepancy can be attributed to the retention of insertions which is an intended feature of HIVGenoPipe. We have two discrepancies of this type within the 30 samples at the 15% ambiguity threshold (Fig. 5, Figure S2). The insertions called by HIVGenoPipe were visible in IGV for the original HyDRA alignment (Fig. 5B, Figure S2B) as well as the alignment of HyDRA reads to the HyDRA consensus at the 15% ambiguity threshold using BWA-MEM for each sample (Fig. 5D, Figure S2D). Since HIVGenoPipe included the inserted nucleotide in the consensus, we could clearly see some reads missing the nucleotide (Fig. 5A and C, Figure S2A and S2C) and we could calculate the ratio of the reads with the inserted nucleotide based on the alignment to the SAMtools consensus sequences (Fig. 5A, Figure S2A) and generate the consensus based on the minor frequency threshold. Finally, we found that the assembler affected the variant frequency for two mismatches, in addition to the effect of the reference sequences: Sample 20 (HXB2 position 2306) and Sample 17 (HXB2 position 3423) (Figures S3 and S4). For the Sample 20 mismatch, the alignment of HyDRA reads to the final consensus sequences from the HIVGenoPipe and HyDRA pipelines using BWA-MEM showed very similar variant frequencies and mapped read counts to each other and to the HIVGenoPipe alignment which would lead to all having the same ambiguous base call of R at the 15% threshold (Figure S3A, S3C, and S3D). In contrast, aligning the HyDRA reads to HXB2 with Bowtie2 has a different minor variant of G at a frequency of 13%, leading to a base call of A (Figure S3B), showing the effect of the reference sequence in the base calling. Aligning the HyDRA reads to HXB2 with BWA-MEM (Figure S3E) or aligning the HyDRA reads to the HyDRA final consensus with Bowtie2 (Figure S3F) can increase the minor variant G frequency to 17% and 18%, respectively, and while not to the level of the HIVGenoPipe frequency (22%), the same ambiguous base call (R) as HIVGenoPipe, at the 15% ambiguity threshold, is made. For the Sample 17 mismatch, the alignment of HyDRA reads to the final consensus sequences from the HIVGenoPipe and HyDRA pipelines using BWA-MEM showed very similar variant frequencies and mapped read counts to each other and to the HIVGenoPipe alignment which leads to all having the same ambiguous base call of R (Figure S4A, S4C, and S4D) at the 15% threshold. Changing either the aligner (Figure S4E) or reference sequence (Figure S4F) is not enough to make the final ambiguous call because the minor variant frequency at this position is on the borderline. Out of 65 discrepancies, only one (Sample 30, HXB2 position 2,307) was not in favor of the HIVGenoPipe result (Fig. 6): the HIVGenoPipe alignment to the intermediate SAMtools consensus sequence showed a minor variant A at this position at a frequency of 14.3% which increased to 15.3% after the mapped reads were filtered with Pysamstats (Fig. 6A); the HyDRA alignment to the HXB2 reference had a minor variant A at this position with a frequency of 9% (Fig. 6B); Using BWA-MEM, HyDRA reads aligned to the final consensus (15% ambiguity threshold) from HIVGenoPipe (Fig. 6C) and from HyDRA (Fig. 6D) indicating the frequency of minor variant A at this position was about 14.35% and 11.06%, respectively, which did not reach the 15% ambiguity threshold, thereby favoring the results from HyDRA. Interestingly, the alignment of HyDRA reads to the HXB2 reference with BWA-MEM also had a minor variant A frequency of 14% at this position (Fig. 6E) and the alignment of HyDRA reads to the HyDRA final consensus with Bowtie2 had a minor variant A frequency of 9% at this position (Fig. 6F).

At the 5% ambiguity threshold, there were 125 mismatches between the consensus sequences of the 30 samples from HIVGenoPipe and HyDRA when we compared them with BLAST. No difference was found in drug resistance reported from the two pipelines (Supplemental Data 4). The causes of these discrepancies were the same as those at the 15% ambiguity threshold: 79 mismatches were due to the reference sequence used in the alignment (Figure S5), 58 mismatches are located at positions where the minor variant frequency is borderline, where small differences in filtered mapping reads between the two pipelines caused slight shifts in the minor variant frequency, pushing the frequency just above or below the threshold (Figure S6). Among these, 14 mismatches were caused by both reference sequences used in the final alignment and frequencies hovering at the borderline (Supplemental Data 4). Finally, there were two discrepancies found in two of the 30 samples caused by insertions retained by HIVGenoPipe at the 5% ambiguous threshold: Sample 3 at HXB2 position 3667 and Sample 14 at HXB2 position 2,379 (Figure S7). We did not find mismatches caused by the aligner choice at this ambiguity threshold.

At the 2% ambiguity threshold, there were 316 mismatches between the 30 samples from.

HIVGenoPipe and HyDRA when we compared them with BLAST. Notably, for Sample 5, 100 mismatches were reported. With the required 10 read minimum for a variant call, at a 2% frequency threshold, we require 500 × coverage for any position of interest to pass the quality threshold. HIVGenoPipe alignment statistics for this sample showed that in a 4490 length alignment, only 1008 positions (~ 22%) passed the quality threshold. These failing positions led to a high number of mismatches in this sample. The consensus sequence of this sample was labeled with a “QUALITY_FAIL” prefix in the file name and a “FAIL” postfix in the sequence ID (Supplemental Data 2). Other samples had between 0 and 22 mismatches, which we considered to be a reasonable increase, compared with the number at 5% and 15% thresholds, given the lower frequency cutoff (Table S4, Supplemental Data 3, 4). The causes for these mismatches were similar to those discussed at the 5% and 15% thresholds. As expected, the insertions retained in Samples 3 and 14 were also present at the 2% threshold. One difference in drug resistance was reported in Sample 23 (HXB2 position 2871) NNRTI ~ RT ~ V108VI, which was characterized by HIVGenoPipe with the ambiguous base (R), but not by HyDRA (G). Using the sample-specific reference, alignment in HIVGenoPipe brought the minor variant frequency over 2% (Figure S8. A), while using a generic reference sequence (HXB2) in HyDRA resulted in a minor variant frequency under 2% (Figure S8. B). Using the final consensus sequences from both pipelines as alignment references resulted in the same minor variant frequencies, over 2% (Figure S8. C, D).

In summary, the consensus sequences generated with HIVGenoPipe are more accurate than those generated with HyDRA due to use of the real-time sample-specific reference sequences in the final alignment. At the 15% ambiguity threshold, a total of 34 are in favor of HIVGenoPipe. Only one borderline mismatch is in favor of HyDRA and the other 30 borderline mismatches could go either way. At the 5% and 2% ambiguity thresholds, we observed the same pattern.

### Result of HIVGenoPipe comparison to Sanger sequences

**Fig. 7:**
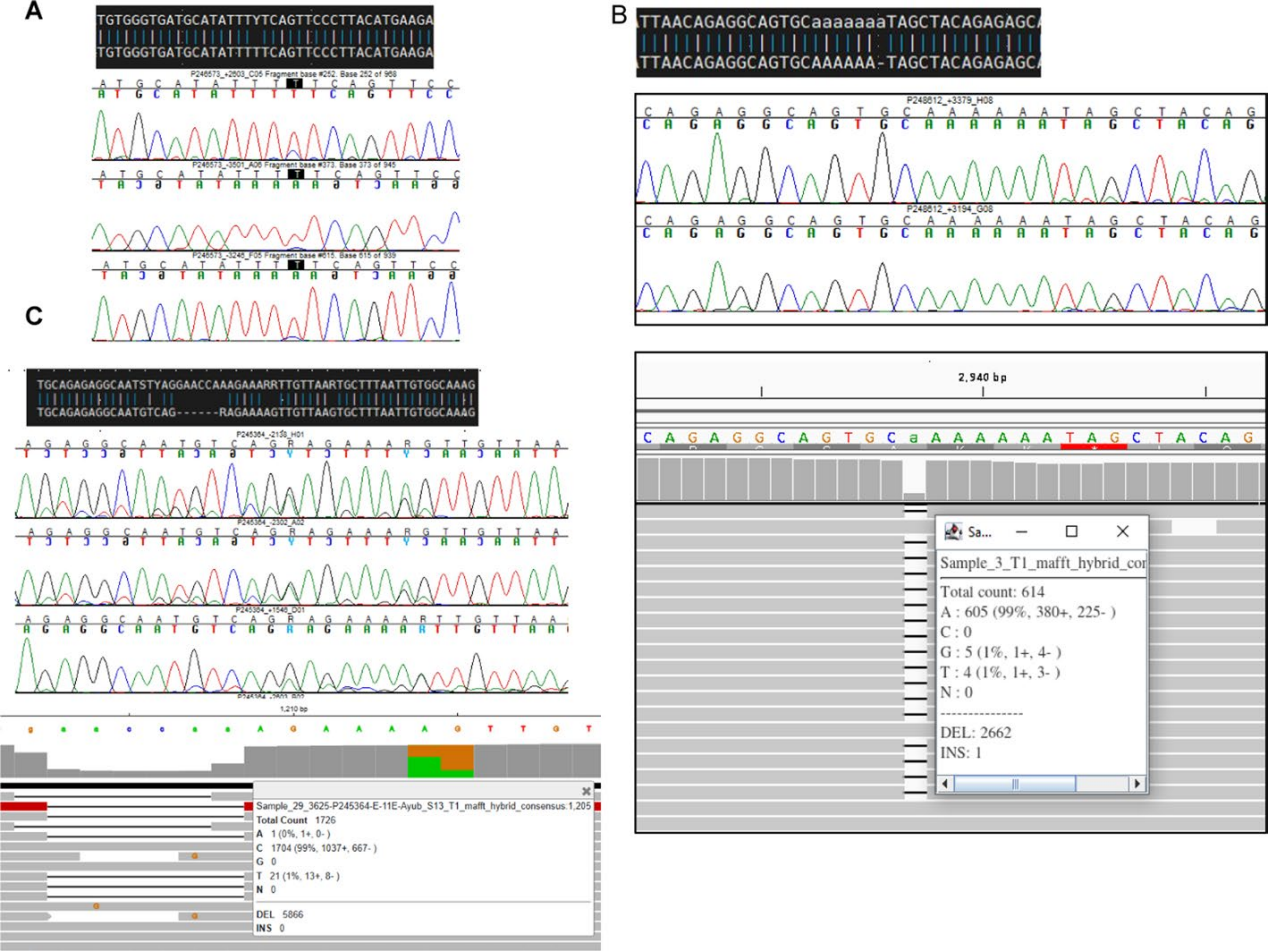
The discrepancy between HIVGenoPipe consensus at 15%ambiguity threshold and Sanger sequencing results. **A** Ambiguous base is visible but not called by Sanger sequencing. **B** HIVGenoPipe retains minor insertions if the frequency is over the ambiguity threshold. (C) HIVGenoPipe retains minor insertions if the frequency is over the ambiguity threshold.The insertion caused low quality bases in the flanking region

To further validate our results, we compared our ambiguous sequences at the 15% ambiguity threshold with the sequences generated using the Sanger sequencing method. Since HyDRA only focuses on ~ 2.8 Kb of the *pol* region, using the Sanger sequences would also validate that the HIVGenoPipe consensus sequences are constructed correctly for the remainder of the sequenced amplicon. In a BLAST comparison of the consensus sequences, 947 discrepancies were identified (Supplemental Data 5). Among them, 853 were the result of the ambiguous bases not being called by the Genetic Analyzer or Sequencher (Fig. 7A); 22 discrepancies were due to insertions or deletions since HIVGenoPipe retains minor insertions if the frequency is over the ambiguity threshold (Fig. 7B and C); 57 discrepancies were caused by the low quality of the Sanger sequences in the region (Fig. 7C, Figure S9). There were two main causes of the low quality in our samples: located at the end of the sequence, which is normal due to the limitation of Sanger sequencing (Figure S9), and the existence of quasispecies with indels in a sample, which creates ambiguous peaks after the indel (Fig. 7C). Minor indel quasispecies have a lesser effect on Sanger sequence quality at low frequencies (Fig. 7B). Finally, we found 19 discrepancies that were not caused by the three reasons above (Supplemental data 5 Column Other). Among them, 11 were caused by a nearby insertion and one was caused by minor quasispecies. We could not find any reason for the other seven nucleotide discrepancies between Sanger sequence assemblies and HIVGenoPipe consensus sequences which represented about 0.005% of the sequences from all tested samples. The HIVGenoPipe consensus sequences at these positions have strong support based on the Illumina sequencing reads, and Sanger sequence assemblies also have Sanger sequencing read support. All seven of these positions are located within the *pol* region and are identical between outputs from HyDRA and HIVGenoPipe.

In summary, the consensus sequences generated by HIVGenoPipe are very accurate, having 99.995% agreement with Sanger consensus sequences from 30 test samples. This is consistent with positive control results in which we know the true sequence: error rates at 2%, 5% and 15% ambiguity thresholds are all 0% (Table S3).

## Discussion

We have developed HIVGenoPipe to detect drug resistance variants in HIV samples tested with NGS reads generated from Illumina MiSeq sequencing. Illumina sequencing is widely used for HIV drug resistance analysis due to its low cost and high accuracy. Since sequencing data generated with other Illumina sequencing machines have the same characteristics, any Illumina sequencing data can be used in this pipeline. In theory, the NGS sequencing data from other platforms can also be used in this pipeline with some modification to accommodate quality control of the other types of instruments. Nextflow was chosen as the platform for the pipeline because it has several advantages: (1) Modularity and reusability: each component is independent and can be easily added to the pipeline. (2) Reproducibility: It supports Docker and Singularity container technologies, making the pipelines reproducible in any Unix/Linux environment. (3) Extensive Tool Integration: Nextflow supports common bioinformatics tools and integrates with container ecosystems for efficient and reproducible analysis. (4) Portability: Nextflow can be executed on multiple platforms without modifying the code. (5) Scalability: Nextflow takes care of parallelizing processes without having to write additional code. The resulting applications are inherently parallel and can scale-up or scale-out transparently (Di Tommaso, et al., 2017). Due to the modular nature, it is easy to update individual tools used in the pipeline if needed.

In HIVGenoPipe, a hybrid reference sequence is generated by ordering de novo assembled contigs and filling in any gaps with the commonly used HXB2 *gag-pol* genome reference sequence. The sequencing reads are aligned to this hybrid reference sequence to generate an intermediate SAMtools consensus sequence. As a result, this intermediate consensus sequence includes insertions even if they only exist in minor quasispecies. This is essential to obtain indel information at user-defined ambiguity thresholds. This consensus sequence is a better representation of the sample sequence than the commonly used HXB2 reference and is used as the reference sequence in the final alignment step to calculate accurate variant frequencies used to generate the final consensus sequences. Our data demonstrate that using a sample-specific reference sequence avoids bias introduced by reference-based assembly and generates more accurate variant frequencies and produces more accurate consensus sequences. The accuracy of the consensus sequences have been validated with the results from the well-accepted pipeline, HyDRA, and Sanger consensus sequences. It was also demonstrated by the positive control test results (Table S3): error rates at 2%, 5% and 15% ambiguity thresholds are all 0%. With accurate consensus sequences, HIVGenoPipe can produce accurate DRM report for users.

Interestingly, we observed that most nucleotide mutations did not lead to drug resistance calls. There are several reasons: (1) Some mutations are located in the non-coding region. (2) Some variants are synonymous mutations. (3) Most non-synonymous mutations do not confer drug resistance since the amino acid change at the given position does not affect either the protein function or drug-protein interaction.

We demonstrated that our pipeline improves alignment accuracy via sample-derived references; however, we recognize that accurate quantification of low-frequency variants remains limited by upstream methods. Zhou and Swanstrom (2020) point out that accurate estimation of minority variant frequencies without UMI-based approaches is limited due to the inability to determine the actual sampling depth of the viral population and the effects of PCR and sequencing errors [44]. However, HIVGenoPipe and HyDRA can accept template consensus sequences derived from UMI-based methods, allowing for more accurate variant frequencies when available.

Even though HIVGenoPipe was only tested and validated for the HIV-1 *gag-pol* region, the pipeline should be compatible with other regions or the full HIV-1 genome by changing the reference input file. Of course, the extended usage needs further testing and validation. Moreover, the novel hybrid assembly procedure developed in this work should be useful for the assembly of similar small genomes.

HIVGenoPipe was designed to be used on private servers by intermediate bioinformatics users in clinical settings. The advantages of this are that the workflow is highly customizable for experienced users and data are kept on local servers. Conversely, a limitation of this design is that HIVGenoPipe is not as easily accessible as web-based applications, such as HyDRA, and requires consideration of hardware, software, and installation. Another limitation is the deidentified data upload to the Stanford HIVdb, which requires internet connectivity. A future feature consideration is the addition of an offline database option to further minimize external data exchanges and improve reproducibility.

In summary, we developed HIVGenoPipe for the detection of HIV-1 drug resistance. The pipeline constructs accurate *gag-pol* consensus sequences using a real-time sample-specific reference sequence generated by a novel hybrid assembly procedure, leading to improved detection of DRMs.

## Availability and requirements

**Project home page:** https://github.com/LHRI-Bioinformatics/HIVGenoPipe.

**Operating system:** Linux.

**Programming language:** Groovy, Python.

**Other requirements:** Nextflow. Choice of container (e.g. Docker).

**License:** the MIT license.

**Any restrictions to use by non-academics:** None.

## Supplementary Information

Additional file 1.

Additional file 2.

## Data Availability

The raw MiSeq sequencing data have been submitted to NCBI SRA under accession number: BioProject PRJNA1218827. The Sanger sequencing assemblies are provided in FASTA format in Supplemental Data 1.

## Abbreviations

AIDS: The acquired immunodeficiency syndrome
ART: Antiretroviral therapy
DRMs: Drug resistance mutations
HIV-1: Human immunodeficiency virus-1
HIVdb: HIV drug resistance database
IN: Integrase
indels: Insertions or deletions
INSTI: Integrase strand transfer inhibitor
NGS: Next generation sequencing
NNRTI: Non-nucleoside reverse transcriptase inhibitor
NRTI: Nucleoside reverse transcriptase inhibitor
PI: Protease inhibitor
PLWH: People/persons living with HIV
PR: Protease
RH: RNase H
RT: Reverse transcriptase
SEAL: Sequence Expression AnaLyzer
WHO: World Health Organization
