# Supplementary material for: HIVGenoPipe: a nextflow pipeline for the detection of HIV-1 drug resistance using a real-time sample-specific reference sequence
Source: BMC Bioinformatics. 2025 Jul 7;26:168. doi: 10.1186/s12859-025-06201-5 (PMC12235847; doi:10.1186/s12859-025-06201-5)
Supplement: Supplementary file 2 — Additional file 2. [file 12859_2025_6201_MOESM2_ESM.docx]

**HIVGenoPipe: a Nextflow Pipeline for the Detection of HIV-1 Drug Resistance Using a Real-Time Sample-Specific Reference Sequence**

Lynn Dotrang^1†^, Brad T. Sherman^1†^, Lisheng Dai^2^, Muhammad Ayub Khan^2^, Helene C. Highbarger^2^, Whitney Bruchey^1^, Sylvain Laverdure^1^, Michael W. Baseler^3^, Tomozumi Imamichi^1^, Robin Dewar^2^, and Weizhong Chang^1^*

# **Supplemental Materials**

## **Method**

## **PCR amplification for HIV gag-pol region from plasma of HIV patient**

HIV viral RNA was extracted from plasma using the EZ1 Advanced XL instrument and the EZ1 Virus Mini Kit v2.0 (Qiagen). The extracted RNA was used for amplification of the full-length HIV gag-pol region (~4,58 Kb) through one-step reverse transcription PCR (RT-PCR) with the PrimeScript™ One Step RT-PCR Kit v2 (Takara) and HIV-specific primers 5'-GCGRCTGGTGAGTACGCC and 5'-CCTAGTGGGATGTGTACTTCTGAAC. The RT-PCR was conducted in three steps: an initial step at 60 °C for 1 hour and 94 °C for 2 minutes; followed by 60 cycles at 94 °C for 1 minute, 61 °C for 30 seconds, and 72 °C for 5 minutes; and a final extension at 72 °C for 10 minutes. The quality of the PCR products was evaluated using agarose gel electrophoresis. The amplified products were then purified and quantified with the Qubit™ dsDNA Assay Kit (Thermo Fisher, Waltham, MA, USA).

## **Next-generation sequencing (NGS) with MiSeq**

Sequencing library preparation and normalization were performed using the Nextera XT DNA Library Preparation Kit and Nextera XT Index Kit (Illumina). Briefly, 1 ng of purified amplicons underwent enzymatic tagmentation, which fragmented the DNA and added partial adapter sequences to the ends of the amplicons. Indexing PCR was subsequently performed to incorporate index and adapter sequences required for cluster generation on the flow cell during sequencing. The resulting NGS libraries were purified, normalized, and treated with NaOH, followed by equimolar pooling. The libraries were then loaded onto a MiSeq Reagent Nano Kit v2 cartridge (500 cycles), and sequencing was conducted on the Illumina MiSeq instrument according to the manufacturer’s standard protocol.

## **Sanger Sequencing**

50ng of amplicon DNA for each sample was used for Sanger sequencing reaction using the BigDye Terminator v3.1 Cycle Sequencing Kit with 3500xL Genetic Analyzer (Thermo Fisher Scientific, Waltham, MA, USA) according to manufacturer’s instructions. The primers were listed in **Supplemental Table 2**. The primer was designed based on HIV-1 strain HXB2 sequence (NCBI Accession: K03455.1) and the name comprise of chain and start position of the primer.

**Supplemental Tables:**

**Table S1:** List of Samples Used for Validation.

| **Sample ID** | **Draw.Date** | **VL_c/mL** | **Read Pair Count** |
| --- | --- | --- | --- |
| Sample 1 | June 18, 2024 | 11,253 | 163,083 |
| Sample 2 | July 11, 2024 | 918,760 | 110,129 |
| Sample 3 | June 11, 2024 | 23,525 | 47,130 |
| Sample 4 | April 3, 2024 | 1,386 | 41,040 |
| Sample 5 | April 10, 2024 | 2,064 | 94,216 |
| Sample 6 | April 10, 2024 | 14,040 | 126,311 |
| Sample 7 | March 6, 2024 | 31,246 | 117,634 |
| Sample 8 | March 12, 2024 | 660,627 | 78,343 |
| Sample 9 | March 12, 2024 | 2,645 | 72,404 |
| Sample 10 | March 15, 2024 | 295,842 | 102,900 |
| Sample 11 | March 19, 2024 | 207,691 | 102,652 |
| Sample 12 | March 5, 2024 | 1,588,039 | 127,570 |
| Sample 13 | February 7, 2024 | 2,164,204 | 83,617 |
| Sample 14 | January 3, 2024 | 3,901 | 83,179 |
| Sample 15 | January 10, 2024 | 1,530,582 | 151,590 |
| Sample 16 | January 10, 2024 | 3,021 | 76,445 |
| Sample 17 | January 16, 2024 | 330,425 | 152,830 |
| Sample 18 | January 16, 2024 | 2,512 | 105,465 |
| Sample 19 | January 17, 2024 | 502,948 | 141,045 |
| Sample 20 | December 7, 2023 | 1,160 | 148,223 |
| Sample 21 | December 13, 2023 | 93,316 | 119,813 |
| Sample 22 | December 14, 2023 | 491,880 | 121,999 |
| Sample 23 | November 9, 2023 | 71,158 | 147,032 |
| Sample 24 | November 15, 2023 | 13,120 | 56,206 |
| Sample 25 | November 21, 2023 | 7,575 | 101,617 |
| Sample 26 | October 26, 2023 | 90,046 | 132,349 |
| Sample 27 | May 26, 2023 | 239,587 | 102,091 |
| Sample 28 | July 26, 2023 | 207,349 | 81,903 |
| Sample 29 | September 25, 2023 | 72,859 | 87,527 |
| Sample 30 | September 28, 2023 | 7,540 | 24,714 |

**Table S2:** List of primers used in Sanger Sequencing of HIV-1 Gag-Pol Amplicon.

| **Primer Name** | **Sequence** |
| --- | --- |
| 796 | GCGAGAGCGTCAGTATTAAGC |
| 1127 | AAAAGGCACAGCAAGCAGCAGCT |
| -1631 | TTTGGTCCTTGTCTTATGTCCAGAATGC |
| 1546 | AATCCACCTATCCCAGTAGGAGAAAT |
| -1960 | CTTTGCCACAATTGAAACACTT |
| 2078 | AGGCTAATTTTTTAGGGA |
| 2165 | CAGAAGAGAGCTTCAGGTTTGGG |
| -2138 | TGTTGGCTCTGGTCTGCTCT |
| -2302 | CTAATAGAGCTTCCTTTAGTTGCC |
| -2603 | GGCCATTGTTTAACTTTTGGG |
| 2603 | CCCAAAAGTTAAACAATGGCC |
| -2691 | TATGGATTTTCAGGCCCAATTTTTGA |
| -2872 | TGCATCACCCACATCCAGTA |
| -3246 | CCATTTATCAGGATGGAGTTC |
| 3194 | CACACCAGACAAAAAACATCAG |
| 3379 | RGCAATTATGTAAACTCCTTAGGGGA |
| -3501 | TAAGTCTTTTGATGGGTCATAATA |
| 3869 | CTATGTAGATGGGGCAGCTA |
| -4176 | TCTACTTGTTCATTTCCTCC |
| 4024 | AAGTAAACATAGTAACAGACTCAC |
| 4162 | CACACAAAGGAATTGGAGGAAATG |
| 4745 | TAAGACAGCAGTACAAATGGCAG |
| -4956 | TACTGCCCCTTCACCTTTCCA |
| -5195 | TAGTGGGATGTGTACTTCTGAAC |

**Table S3:** Example of the statistics for the control samples.

| **Negative Control** |  |  |  |
| --- | --- | --- | --- |
| QC Metric | QC Status | Value | QC Range |
| Sample |  | HivNeg_T1 |  |
| sampleId |  | HivNeg_T1 |  |
| Total HIV Read1 | PASSED | 17 | <=1000 |
| Contig Coverage (%) | PASSED | 0 | 0 |
|  |  |  |  |
| **Positive Control** |  |  |  |
| Sample |  | HivPos_T1 |  |
| sampleId |  | HivPos_T1 |  |
| Total HIV Read1 | PASSED | 90213 | >=5000 |
| Total HIV Read1(% of raw) | PASSED | 78.010584 | 50-100 |
| Median Depth (reads) | PASSED | 10789 | 200-100000000 |
| Depth MAD (reads) | PASSED | 2264.8139365130232 | 200-100000000 |
| Contig Coverage (%) | PASSED | 1 | 100% |
| Error Rate (Read Variation %) | PASSED | 0.002528836 | <1% |
| Contig Error Rate at 1% (%) | PASSED | 0.001406964 | <1% |
| Contig Error Rate at 2% (%) | PASSED | 0 | <0.1% |
| Contig Error Rate at 5% (%) | PASSED | 0 | <0.1% |
| Contig Error Rate at 15% (%) | PASSED | 0 | <0.1% |

**Table S4:** Comparison of final consensus at 2% for HIVGenopipe and HyDRA with BLAST

| **Sample ID** | **BLASTN Identities** | **# of mismatches** |
| --- | --- | --- |
| Sample 1 | 2841/2844 | 3 |
| Sample 2 | 2841/2841 | 0 |
| Sample 3 | 2839/2845 | 6 |
| Sample 4 | 2841/2841 | 0 |
| Sample 6 | 2824/2844 | 20 |
| Sample 7 | 2840/2844 | 4 |
| Sample 8 | 2834/2844 | 10 |
| Sample 9 | 2844/2844 | 0 |
| Sample 10 | 2830/2844 | 14 |
| Sample 11 | 2832/2844 | 12 |
| Sample 12 | 2823/2841 | 18 |
| Sample 13 | 2840/2844 | 4 |
| Sample 14 | 2838/2842 | 4 |
| Sample 15 | 2837/2844 | 7 |
| Sample 16 | 2839/2841 | 2 |
| Sample 17 | 2823/2844 | 21 |
| Sample 18 | 2834/2836 | 2 |
| Sample 19 | 2830/2844 | 14 |
| Sample 20 | 2844/2844 | 0 |
| Sample 21 | 2841/2843 | 2 |
| Sample 22 | 2824/2841 | 17 |
| Sample 23 | 2827/2841 | 14 |
| Sample 24 | 2837/2841 | 4 |
| Sample 25 | 2840/2841 | 1 |
| Sample 26 | 2837/2841 | 4 |
| Sample 27 | 2833/2844 | 11 |
| Sample 28 | 2835/2841 | 6 |
| Sample 29 | 2832/2843 | 11 |
| Sample 30 | 2839/2844 | 5 |
| Sample 5 * | 2741/2841 | 100 |

***Note:** The Sample 5 didn’t pass consensus sequence QC at 2% threshold

**
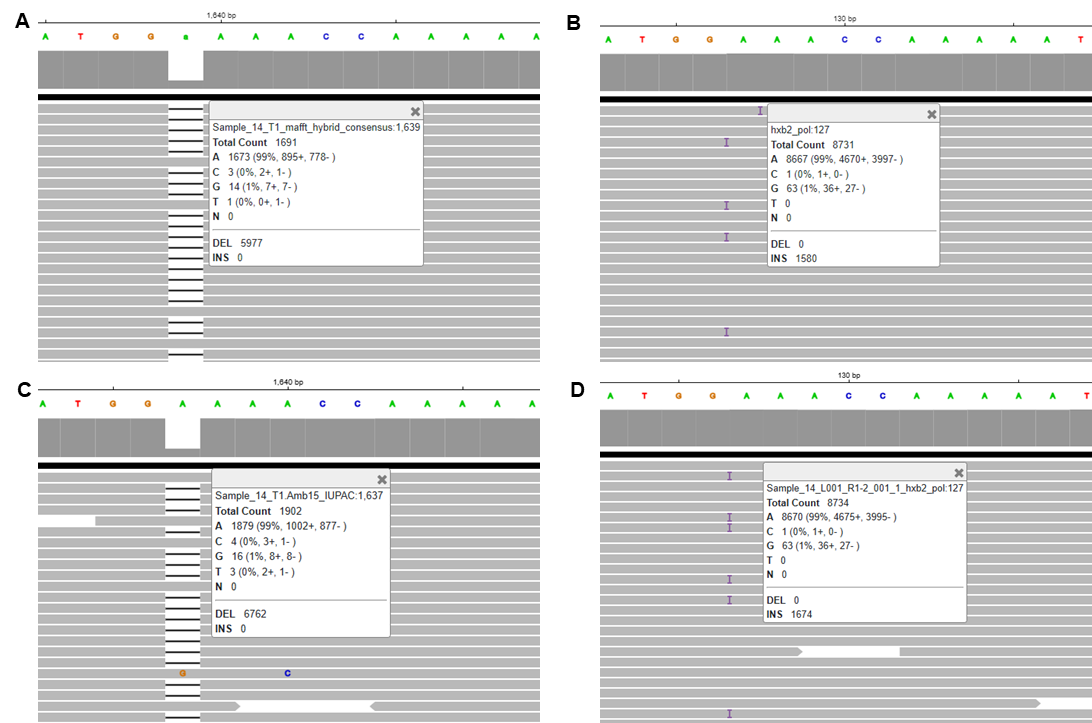

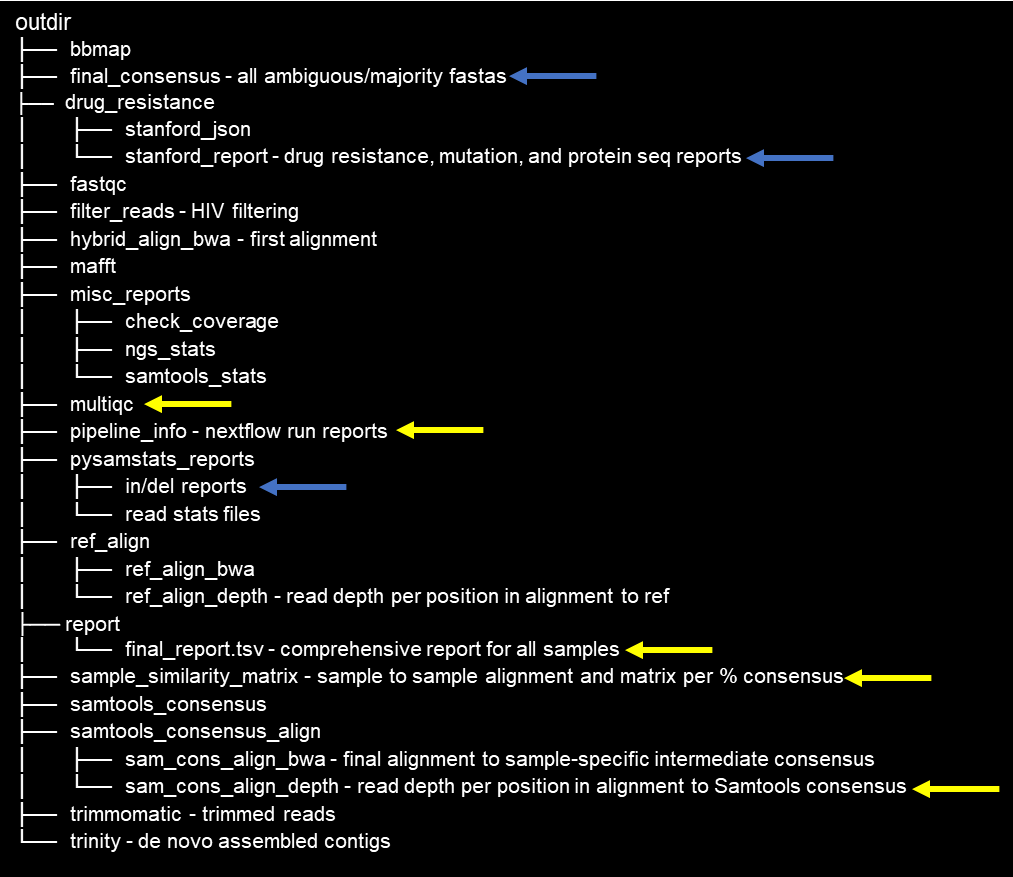
Supplemental Figures**

**Figure S1. Output Directory Structure of HIVGenoPipe.** Each module of HIVGenoPipe will return its output files into its respective directory. The final reporting files for user are indicated by the blue arrow. The important QC files for user to check are indicated by the yellow arrow.

**Figure S2. The position of different base call caused by the inclusion of insertion with minor abundancy in HIVGenoPipe: Sample 14 at HXB2 position 2,379** (corresponding to coordinates on full length HXB2). (A) Final alignment in HIVGenoPipe. Sequencing reads aligned to the intermediate Samtools consensus with BWA (mm p=3). (B) Final HyDRA alignment. HyDRA reads aligned to HXB2 with Bowtie2. (C) HyDRA reads aligned to the HIVGenoPipe final consensus (15% ambiguity threshold) with BWA (mm p=3). (D) HyDRA reads aligned to the HyDRA final consensus (15% ambiguity threshold) with BWA (mm p=3).

**
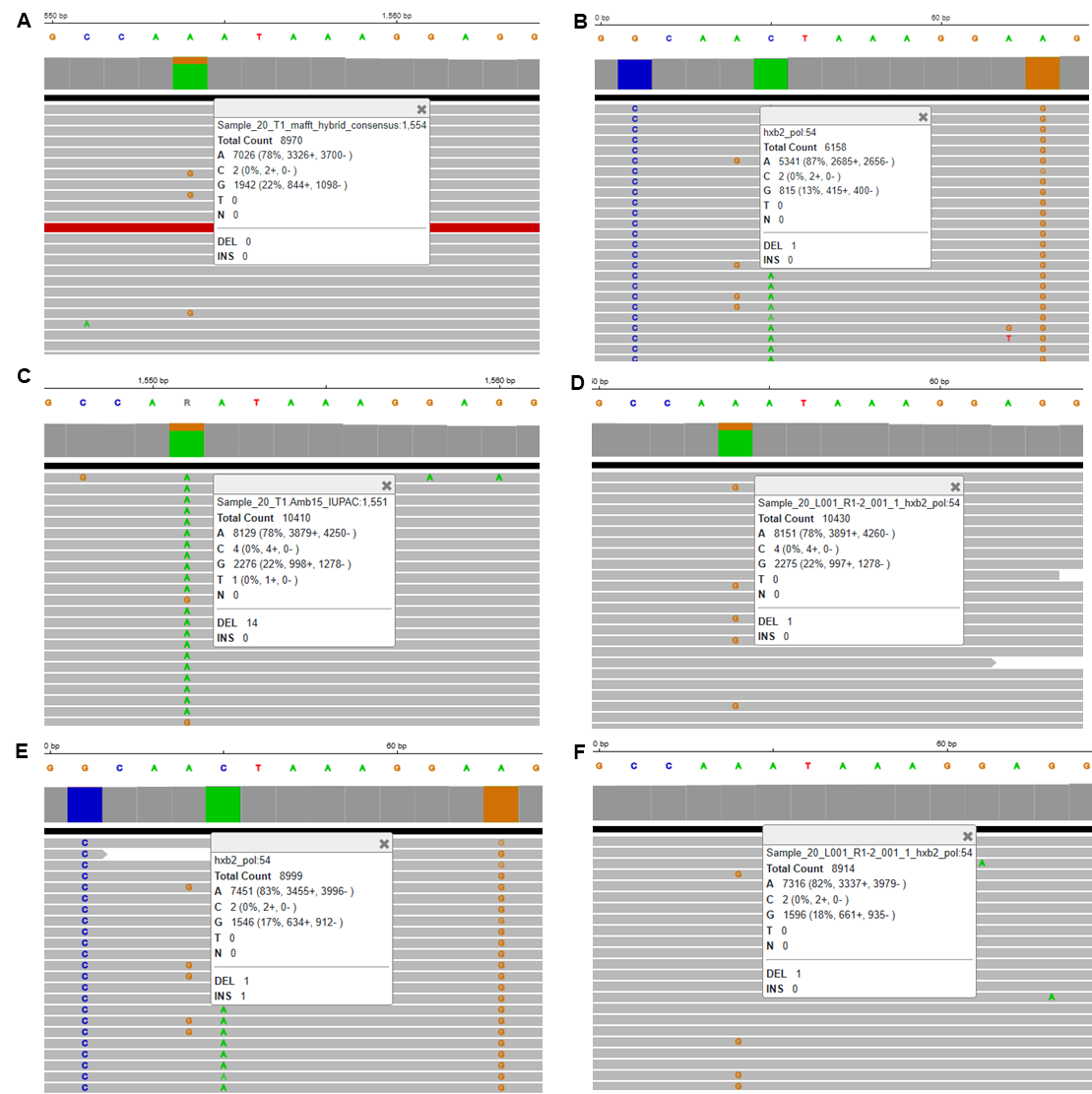
**

**Figure S3. Both Assembler and reference affect ambiguous base call: Sample 20 at HXB2 position 2,306** (corresponding to coordinates on full length HXB2). (A) Final alignment in HIVGenoPipe. Sequencing reads aligned to the intermediate Samtools consensus with BWA (mm p=3). (B) Final HyDRA alignment. HyDRA reads aligned to HXB2 with Bowtie2. (C) HyDRA reads aligned to the HIVGenoPipe final consensus (15% ambiguity threshold) with BWA (mm p=3). (D) HyDRA reads aligned to the HyDRA final consensus (15% ambiguity threshold) with BWA (mm p=3). (E) HyDRA reads aligned to HXB2 with BWA (mm p=3) (F) HyDRA reads aligned to the HyDRA final consensus (15% ambiguity threshold) with Bowtie2.

**
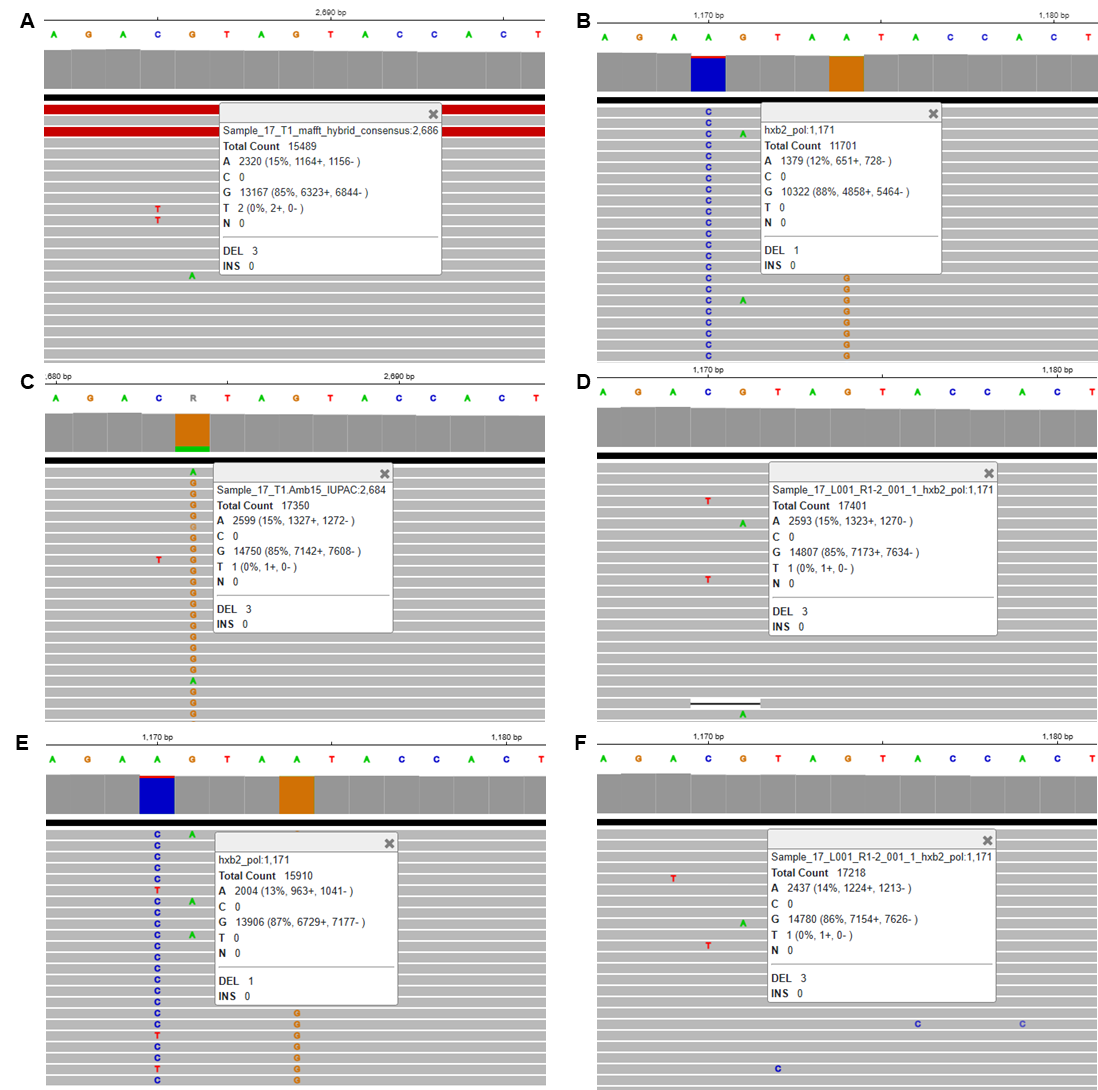
**

**Figure S4. Both Assembler and reference affect ambiguous base call: Sample 17 at HXB2 position 3,423** (corresponding to coordinates on full length HXB2). (A) Final alignment in HIVGenoPipe. Sequencing reads aligned to the intermediate Samtools consensus with BWA (mm p=3). (B) Final HyDRA alignment. HyDRA reads aligned to HXB2 with Bowtie2. (C) HyDRA reads aligned to the HIVGenoPipe final consensus (15% ambiguity threshold) with BWA (mm p=3). (D) HyDRA reads aligned to the HyDRA final consensus (15% ambiguity threshold) with BWA (mm p=3). (E) HyDRA reads aligned to HXB2 with BWA (mm p=3) (F) HyDRA reads aligned to the HyDRA final consensus (15% ambiguity threshold) with Bowtie2.

**
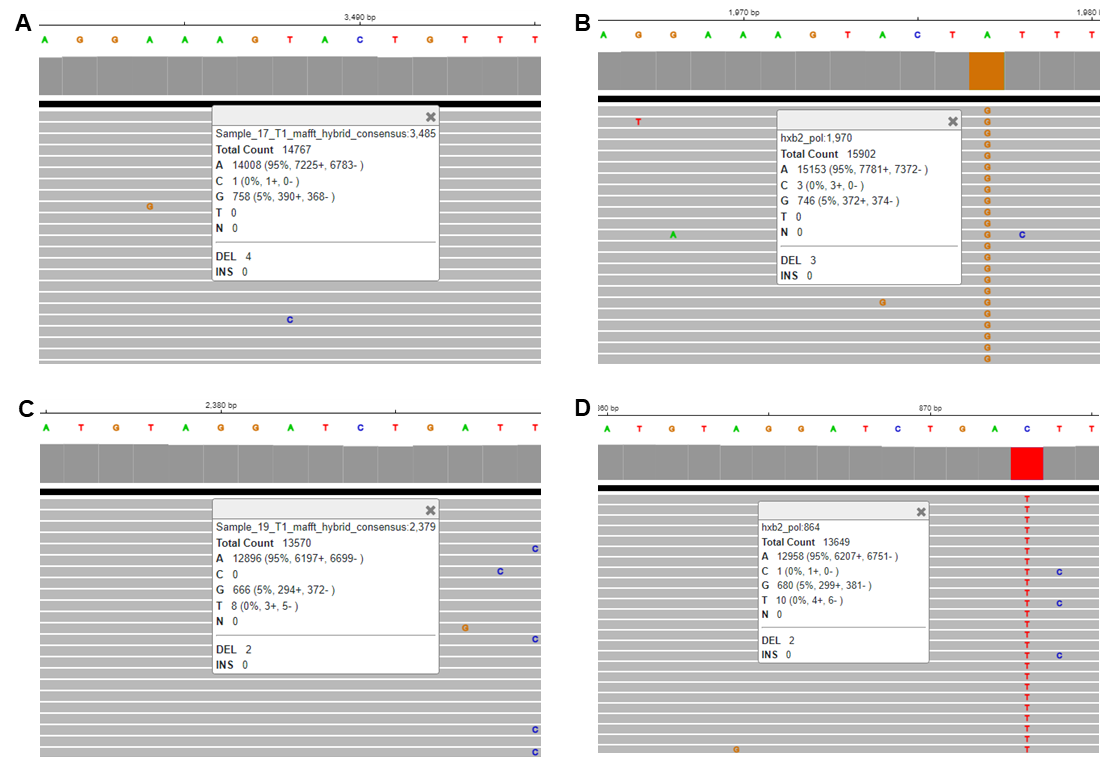

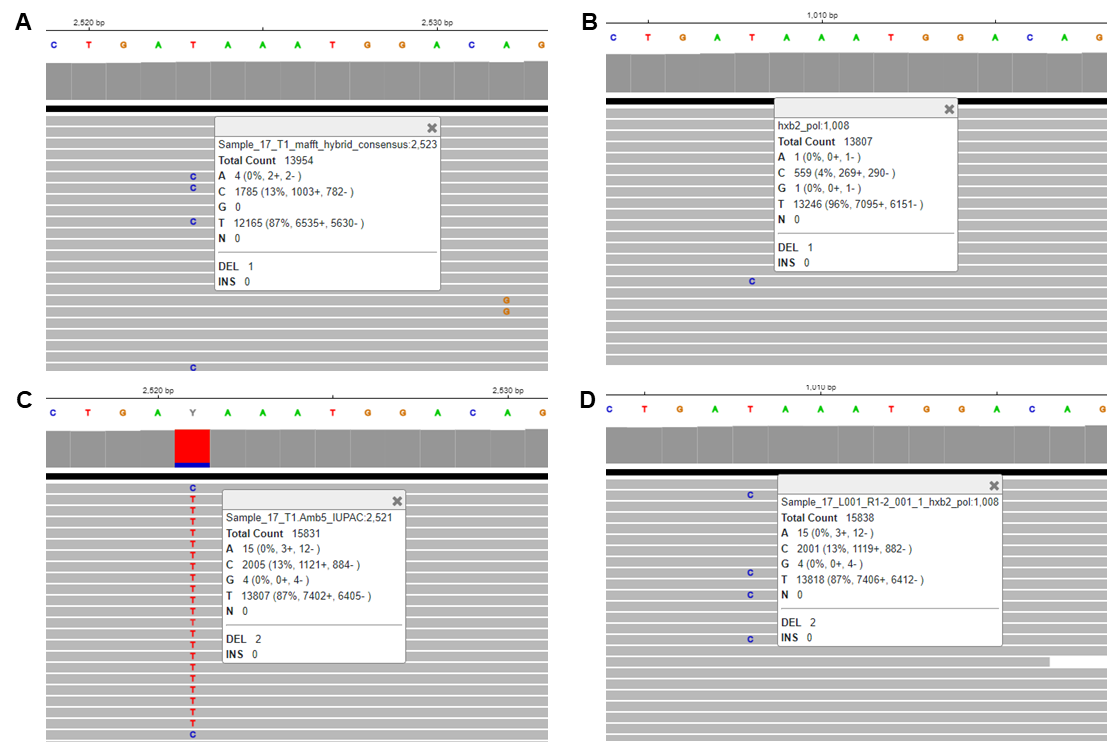
**

**Figure S6. The position with different base call with minor variant frequency at the borderline: Sample 17 at HXB2 position 4,222** (A, B) **and Sample 19 at HXB2 position 3,116** (C, D) (All coordinates correspond to coordinates on full length HXB2). (A, C) Final alignment in HIVGenoPipe. Sequencing reads aligned to the intermediate Samtools consensus with BWA (mm p=3). (B, D) Final HyDRA alignment. HyDRA reads aligned to HXB2 with Bowtie2.

**Figure S5. Sample-specific reference in HIVGenoPipe results in accurate ambiguous base call: Sample 17 at HXB2 position 3,260,** (corresponding to coordinates on full length HXB2). (A) Final alignment in HIVGenoPipe. Sequencing reads aligned to the intermediate Samtools consensus with BWA (mm p=3). (B) Final HyDRA alignment. HyDRA reads aligned to HXB2 with Bowtie2. (C) HyDRA reads aligned to the HIVGenoPipe final consensus (5% ambiguity threshold) with BWA (mm p=3). (D) HyDRA reads aligned to the HyDRA final consensus (5% ambiguity threshold) with BWA (mm p=3).

**
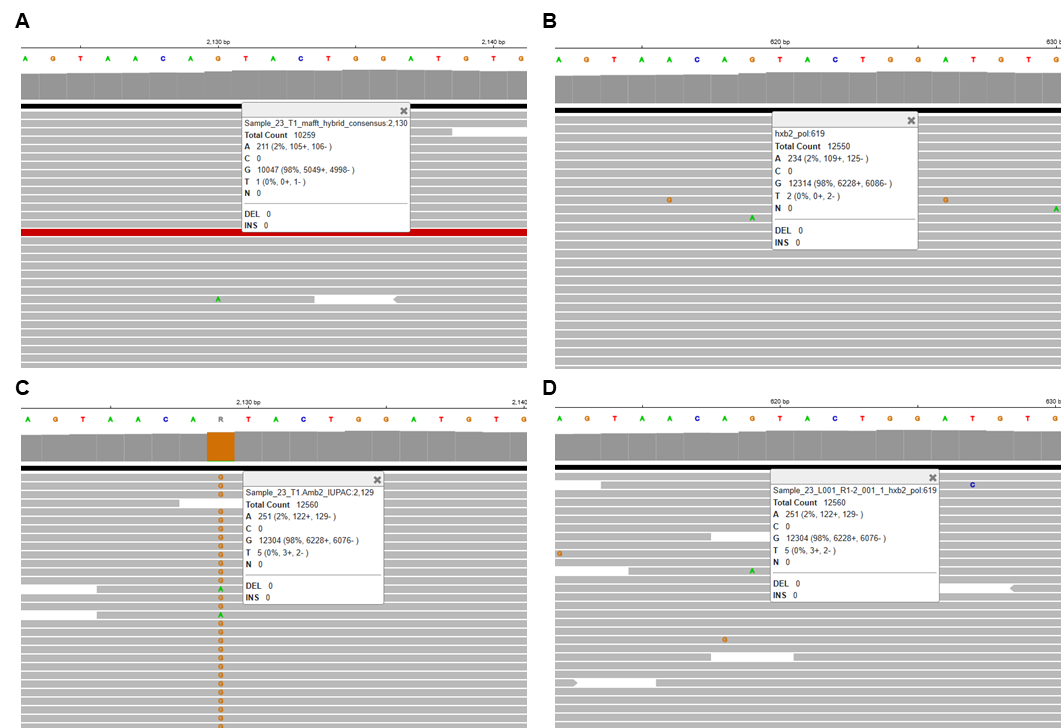

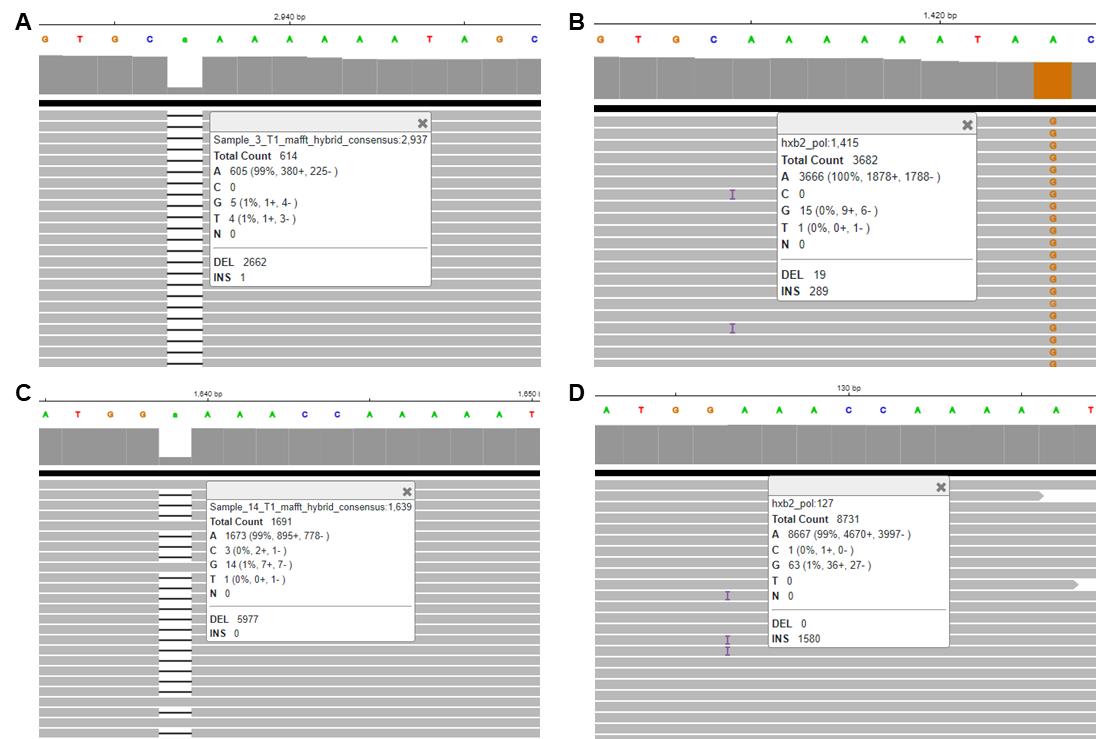
**

**Figure S8. The position with different base call with minor variant frequency at the borderline: Sample 23 at HXB2 position 2,871** (All coordinates correspond to coordinates on full length HXB2). A) Final alignment in HIVGenoPipe. Sequencing reads aligned to the intermediate Samtools consensus with BWA (mm p=3). (B) Final HyDRA alignment. HyDRA reads aligned to HXB2 with Bowtie2. (C) HyDRA reads aligned to the HIVGenoPipe final consensus (2% ambiguity threshold) with BWA (mm p=3). (D) HyDRA reads aligned to the HyDRA final consensus (2% ambiguity threshold) with BWA (mm p=3).

**Figure S7. The position of different base call caused by the inclusion of insertion with minor abundancy in HIVGenoPipe: Sample 3 at HXB2 position 3,667** (A, B) **and Sample 14 at HXB2 position 2,379** (C, D)**,** (All coordinates correspond to coordinates on full length HXB2). (A, C) Final alignment in HIVGenoPipe. Sequencing reads aligned to the intermediate Samtools consensus with BWA (mm p=3). (B, D) Final HyDRA alignment. HyDRA reads aligned to HXB2 with Bowtie2.


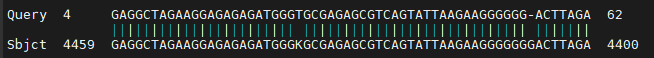


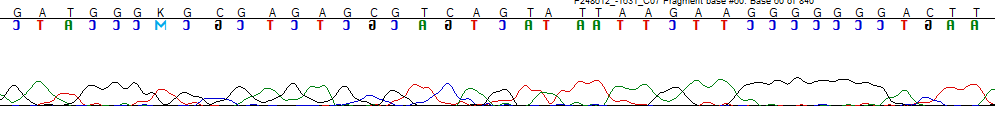


**Figure S9. The base quality of Sanger sequencing degraded at the end of the sequence**.
