# Supplementary material for: HIVGenoPipe: a nextflow pipeline for the detection of HIV-1 drug resistance using a real-time sample-specific reference sequence
Source: BMC Bioinformatics. 2025 Jul 7;26:168. doi: 10.1186/s12859-025-06201-5 (PMC12235847; doi:10.1186/s12859-025-06201-5)
Supplement: Supplementary file 1 — Additional file 1. [file 12859_2025_6201_MOESM1_ESM.zip › Supplemental Data 2-Example_Output.zip!/Supplemental_Data_Example_Output/multiqc/multiqc_plots/pdf/mqc_fastqc_adapter_content_plot_1.pdf]

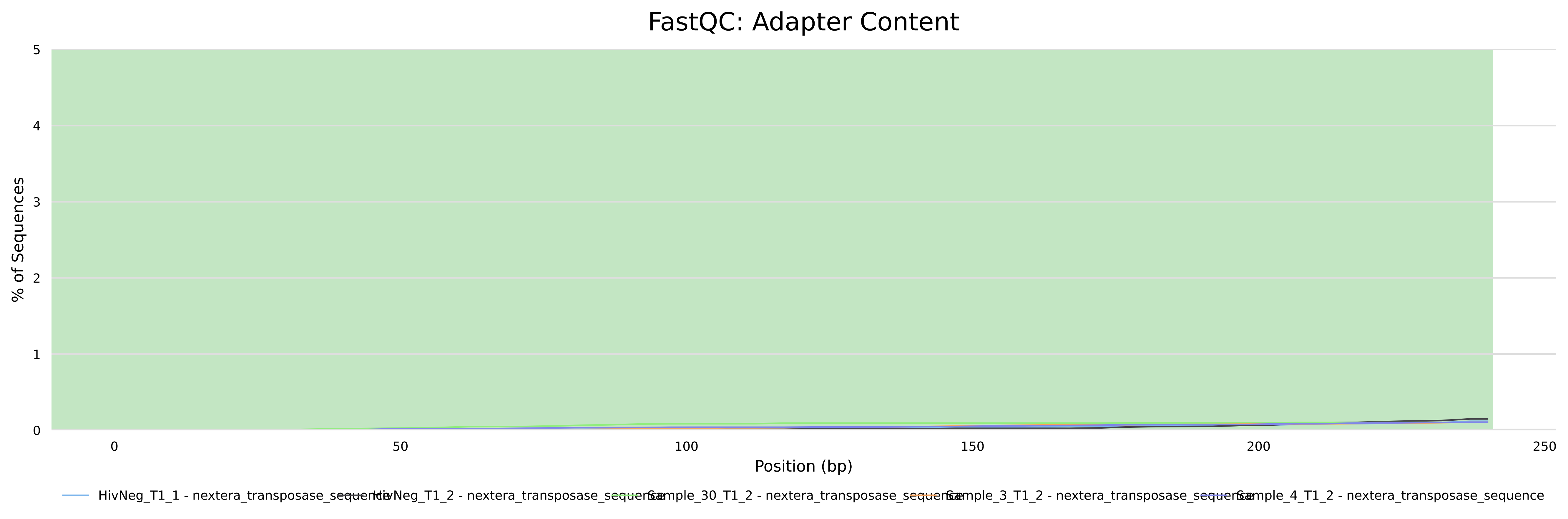
FastQC: Adapter Content
% of Sequences
5
4
3
2
1
0
0
50
100
150
200
250
Position (bp)
HivNeg_T1_1 - nextera_transposase_sequence
HivNeg_T1_2 - nextera_transposase_sequence
Sample_30_T1_2 - nextera_transposase_sequence
Sample_3_T1_2 - nextera_transposase_sequence
Sample_4_T1_2 - nextera_transposase_sequence
