# Supplementary material for: HIVGenoPipe: a nextflow pipeline for the detection of HIV-1 drug resistance using a real-time sample-specific reference sequence
Source: BMC Bioinformatics. 2025 Jul 7;26:168. doi: 10.1186/s12859-025-06201-5 (PMC12235847; doi:10.1186/s12859-025-06201-5)
Supplement: Supplementary file 1 — Additional file 1. [file 12859_2025_6201_MOESM1_ESM.zip › Supplemental Data 2-Example_Output.zip!/Supplemental_Data_Example_Output/multiqc/multiqc_plots/pdf/mqc_fastqc_per_sequence_quality_scores_plot_1.pdf]

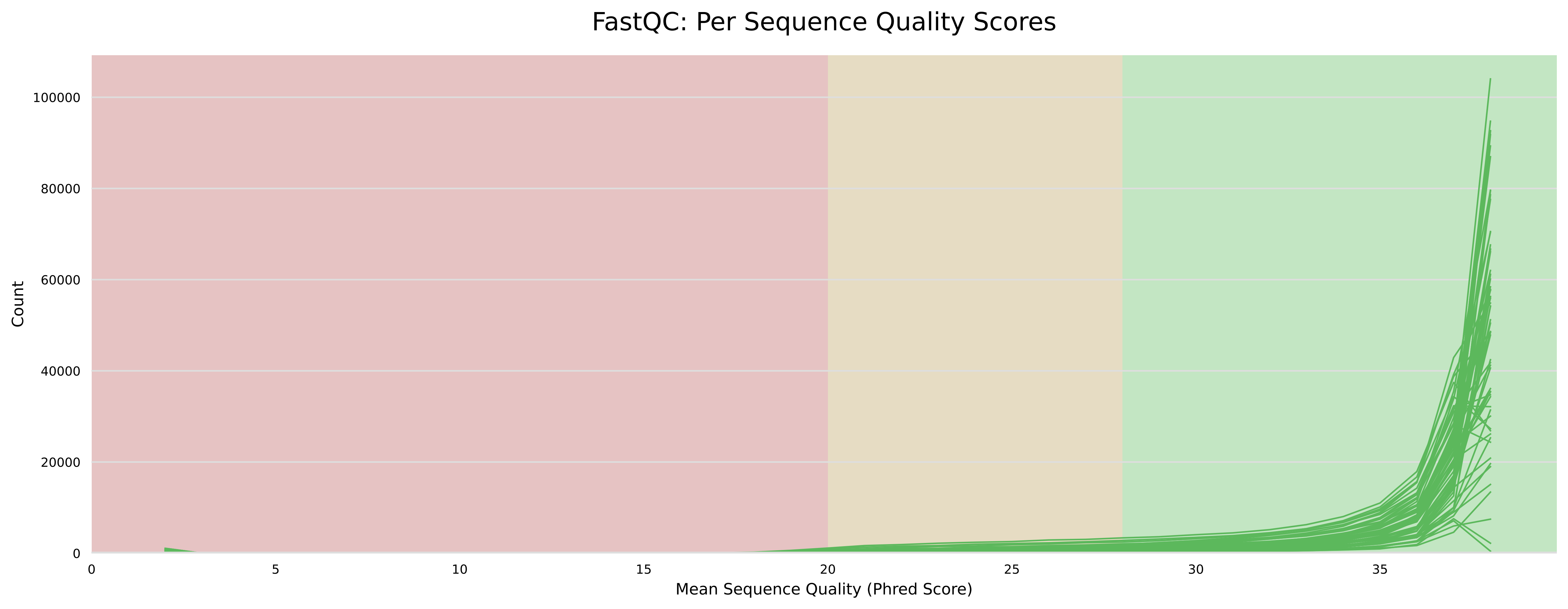
FastQC: Per Sequence Quality Scores
Count
Mean Sequence Quality (Phred Score)
0
20000
40000
60000
80000
100000
0
5
10
15
20
25
30
35
