# Supplementary material for: HIVGenoPipe: a nextflow pipeline for the detection of HIV-1 drug resistance using a real-time sample-specific reference sequence
Source: BMC Bioinformatics. 2025 Jul 7;26:168. doi: 10.1186/s12859-025-06201-5 (PMC12235847; doi:10.1186/s12859-025-06201-5)
Supplement: Supplementary file 1 — Additional file 1. [file 12859_2025_6201_MOESM1_ESM.zip › Supplemental Data 2-Example_Output.zip!/Supplemental_Data_Example_Output/multiqc/multiqc_plots/pdf/mqc_fastqc_sequence_counts_plot_1.pdf]

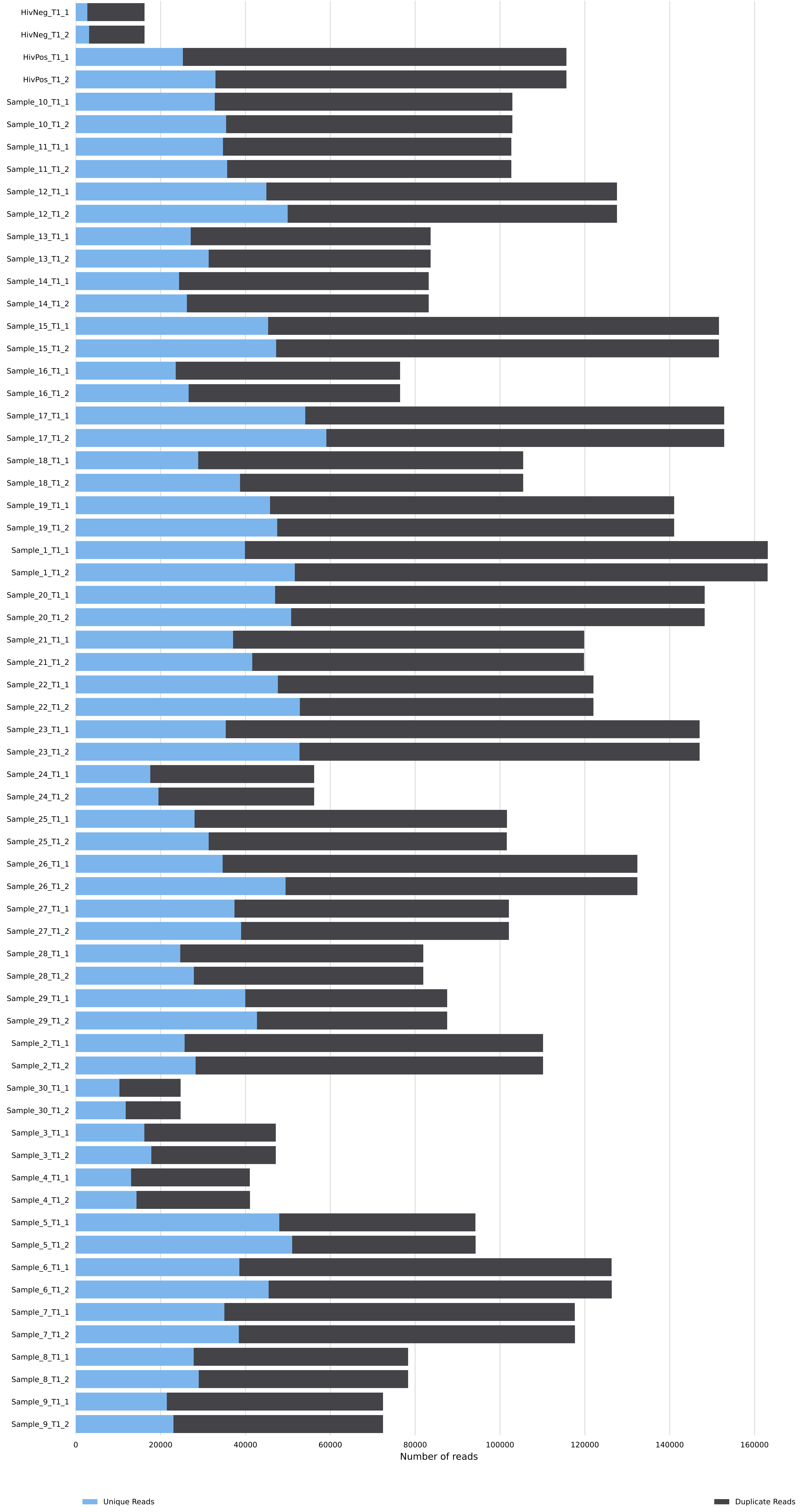
HivNeg_T1_1
HivNeg_T1_2
HivPos_T1_1
HivPos_T1_2
Sample_10_T1_1
Sample_10_T1_2
Sample_11_T1_1
Sample_11_T1_2
Sample_12_T1_1
Sample_12_T1_2
Sample_13_T1_1
Sample_13_T1_2
Sample_14_T1_1
Sample_14_T1_2
Sample_15_T1_1
Sample_15_T1_2
Sample_16_T1_1
Sample_16_T1_2
Sample_17_T1_1
Sample_17_T1_2
Sample_18_T1_1
Sample_18_T1_2
Sample_19_T1_1
Sample_19_T1_2
Sample_1_T1_1
Sample_1_T1_2
Sample_20_T1_1
Sample_20_T1_2
Sample_21_T1_1
Sample_21_T1_2
Sample_22_T1_1
Sample_22_T1_2
Sample_23_T1_1
Sample_23_T1_2
Sample_24_T1_1
Sample_24_T1_2
Sample_25_T1_1
Sample_25_T1_2
Sample_26_T1_1
Sample_26_T1_2
Sample_27_T1_1
Sample_27_T1_2
Sample_28_T1_1
Sample_28_T1_2
Sample_29_T1_1
Sample_29_T1_2
Sample_2_T1_1
Sample_2_T1_2
Sample_30_T1_1
Sample_30_T1_2
Sample_3_T1_1
Sample_3_T1_2
Sample_4_T1_1
Sample_4_T1_2
Sample_5_T1_1
Sample_5_T1_2
Sample_6_T1_1
Sample_6_T1_2
Sample_7_T1_1
Sample_7_T1_2
Sample_8_T1_1
Sample_8_T1_2
Sample_9_T1_1
Sample_9_T1_2
0
20000
40000
60000
80000
100000
120000
140000
160000
Number of reads
Unique Reads
Duplicate Reads
