# Supplementary material for: HIVGenoPipe: a nextflow pipeline for the detection of HIV-1 drug resistance using a real-time sample-specific reference sequence
Source: BMC Bioinformatics. 2025 Jul 7;26:168. doi: 10.1186/s12859-025-06201-5 (PMC12235847; doi:10.1186/s12859-025-06201-5)
Supplement: Supplementary file 1 — Additional file 1. [file 12859_2025_6201_MOESM1_ESM.zip › Supplemental Data 2-Example_Output.zip!/Supplemental_Data_Example_Output/multiqc/multiqc_plots/pdf/mqc_fastqc_sequence_duplication_levels_plot_1.pdf]

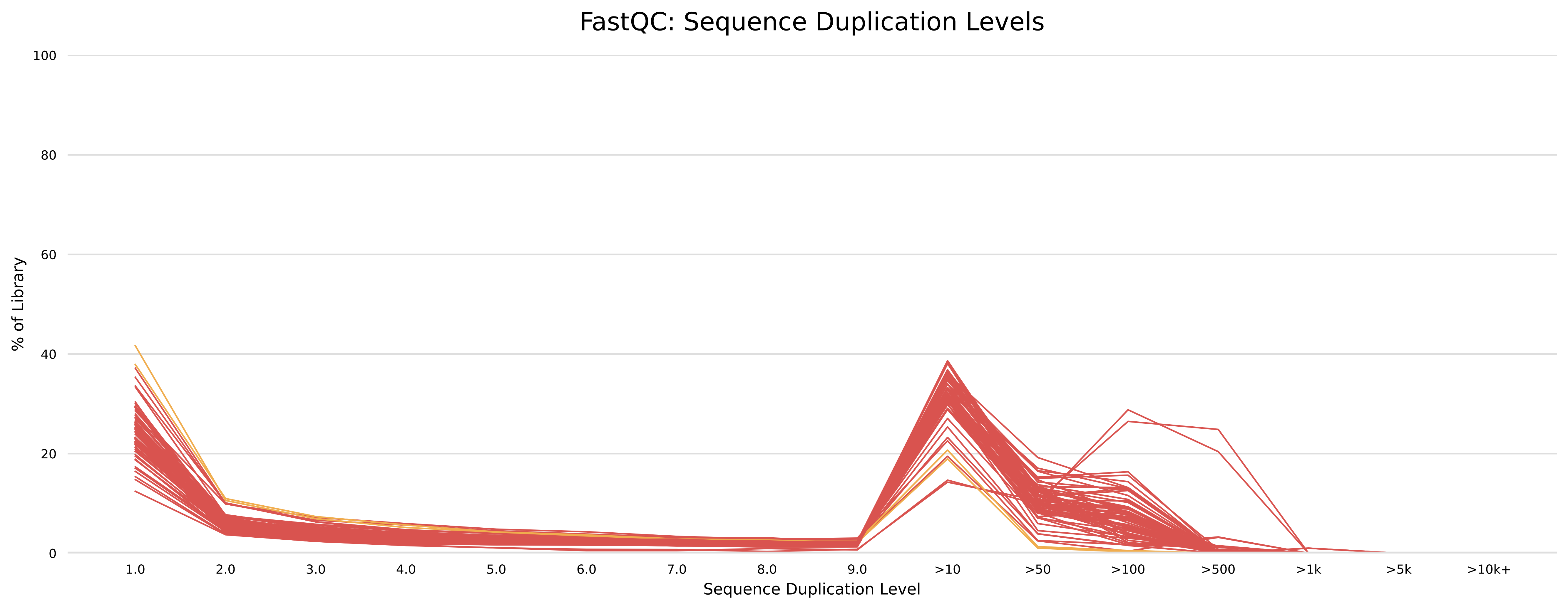

FastQC: Sequence Duplication Levels
100
80
60
40
20
0
% of Library
1.0
2.0
3.0
4.0
5.0
6.0
7.0
8.0
9.0
>10
>50
>100
>500
>1k
>5k
>10k+
Sequence Duplication Level
