# Supplementary material for: HIVGenoPipe: a nextflow pipeline for the detection of HIV-1 drug resistance using a real-time sample-specific reference sequence
Source: BMC Bioinformatics. 2025 Jul 7;26:168. doi: 10.1186/s12859-025-06201-5 (PMC12235847; doi:10.1186/s12859-025-06201-5)
Supplement: Supplementary file 1 — Additional file 1. [file 12859_2025_6201_MOESM1_ESM.zip › Supplemental Data 2-Example_Output.zip!/Supplemental_Data_Example_Output/multiqc/multiqc_plots/pdf/mqc_samtools_alignment_plot_1_pc.pdf]

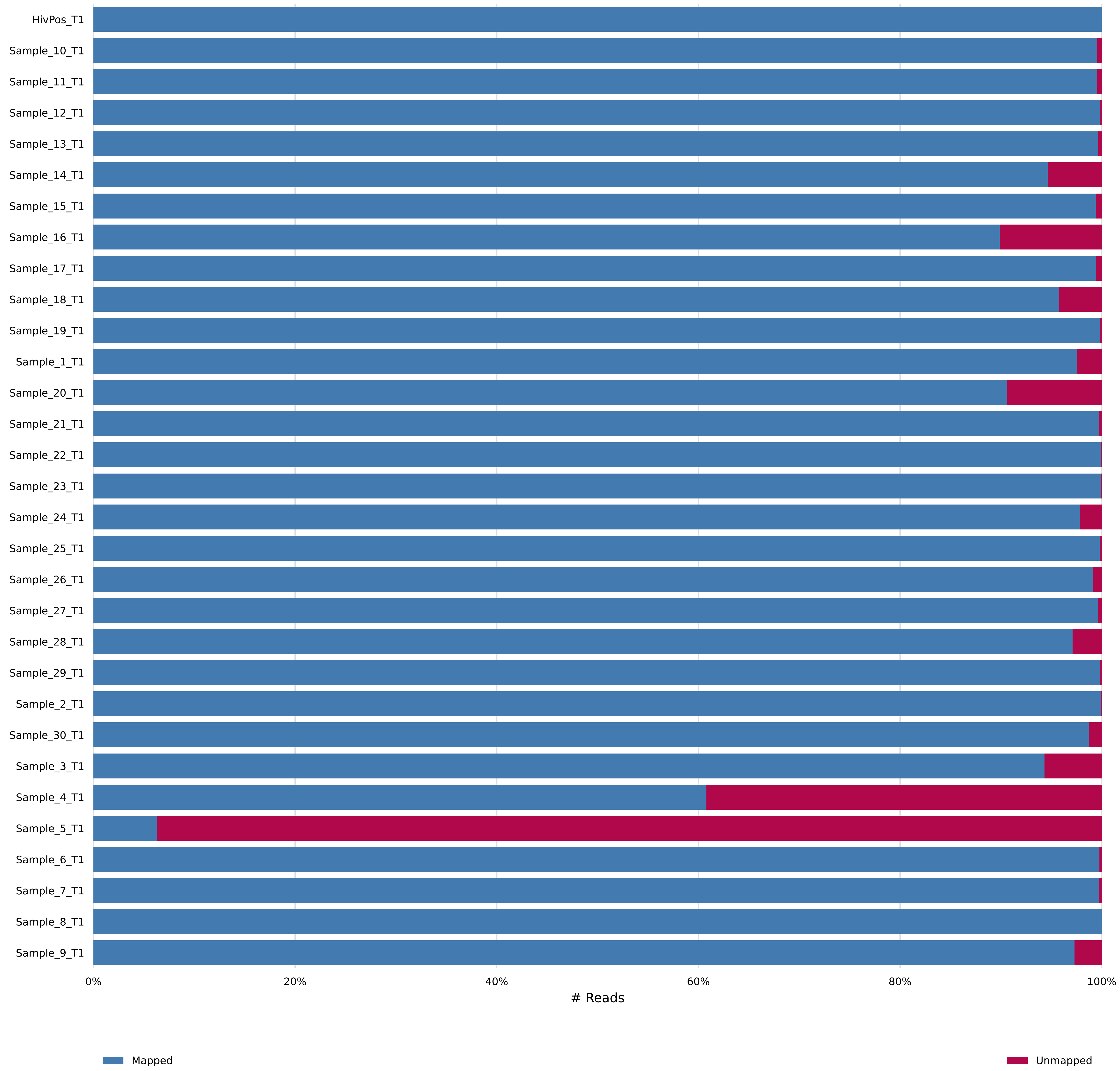
HivPos_T1
Sample_10_T1
Sample_11_T1
Sample_12_T1
Sample_13_T1
Sample_14_T1
Sample_15_T1
Sample_16_T1
Sample_17_T1
Sample_18_T1
Sample_19_T1
Sample_1_T1
Sample_20_T1
Sample_21_T1
Sample_22_T1
Sample_23_T1
Sample_24_T1
Sample_25_T1
Sample_26_T1
Sample_27_T1
Sample_28_T1
Sample_29_T1
Sample_2_T1
Sample_30_T1
Sample_3_T1
Sample_4_T1
Sample_5_T1
Sample_6_T1
Sample_7_T1
Sample_8_T1
Sample_9_T1
0%
20%
40%
60%
80%
100%
Reads
Mapped
Unmapped
