# Supplementary material for: HIVGenoPipe: a nextflow pipeline for the detection of HIV-1 drug resistance using a real-time sample-specific reference sequence
Source: BMC Bioinformatics. 2025 Jul 7;26:168. doi: 10.1186/s12859-025-06201-5 (PMC12235847; doi:10.1186/s12859-025-06201-5)
Supplement: Supplementary file 1 — Additional file 1. [file 12859_2025_6201_MOESM1_ESM.zip › Supplemental Data 2-Example_Output.zip!/Supplemental_Data_Example_Output/pipeline_info/execution_report_2025-05-15_15-27-41.html]

[trusting\_coulomb] Nextflow Workflow Report


Nextflow Report


- Summary
- Resources
- Tasks

[trusting\_coulomb]

# Nextflow workflow report

## `[trusting_coulomb]` *(resumed run)*

#### Workflow execution completed unsuccessfully!

The exit status of the task that caused the workflow execution to fail was: `null`.

The full error message was:

```
SIGINT
```

Run times
:   15-May-2025 15:27:48 - 15-May-2025 15:41:53
    (duration: **14m 5s**)

164 succeeded

2 cached

1 ignored

1 failed

Nextflow command
:   ```
    nextflow run /mnt/LHRI/Bioinformatics/Projects/HIVGenoPipe/github/HIVGenoPipe_Pub/main.nf --input LD_30_Sanger_samplesheet_clean_controls.csv --fasta ../test_data/resources/K03455.1_HXB2_737-5219.fasta --positive_control_ref ../test_data/resources/NL4_3.fasta -profile docker --outdir ./05092025_30_samples_amb2_run -resume
    ```

CPU-Hours
:   `4.7 (0.7% failed)`

Launch directory
:   `/array/Members/dotrangtl/082124_LD_30_Sanger_test`

Work directory
:   `/array/Members/dotrangtl/082124_LD_30_Sanger_test/work`

Project directory
:   `/mnt/LHRI/Bioinformatics/Projects/HIVGenoPipe/github/HIVGenoPipe_Pub`

Script name
:   `main.nf`

Script ID
:   `6c46fa9cd08761b5de27f8fcbed649c4`

Workflow session
:   `2a76e6f1-25a2-4b99-a2fa-51ccc2da19d2`

Workflow profile
:   docker

Nextflow version
:   version 23.10.1, build 5891 (12-01-2024 22:01 UTC)

## Resource Usage

These plots give an overview of the distribution of resource usage for each process.

#### CPU

- Raw Usage
- % Allocated

#### Memory

- Physical (RAM)
- Virtual (RAM + Disk swap)
- % RAM Allocated

#### Job Duration

- Raw Usage
- % Allocated

#### I/O

- Read
- Write

## Tasks

This table shows information about each task in the workflow. Use the search box on the right
to filter rows for specific values. Clicking headers will sort the table by that value and
scrolling side to side will reveal more columns.

Values shown as:

Human readable
Raw values

(tasks table omitted because the dataset is too big)

Generated by Nextflow, version 23.10.1
