# Supplementary material for: HIVGenoPipe: a nextflow pipeline for the detection of HIV-1 drug resistance using a real-time sample-specific reference sequence
Source: BMC Bioinformatics. 2025 Jul 7;26:168. doi: 10.1186/s12859-025-06201-5 (PMC12235847; doi:10.1186/s12859-025-06201-5)
Supplement: Supplementary file 1 — Additional file 1. [file 12859_2025_6201_MOESM1_ESM.zip › Supplemental Data 2-Example_Output.zip!/Supplemental_Data_Example_Output/pipeline_info/execution_report_2025-05-15_15-42-12.html]

[mad\_bell] Nextflow Workflow Report


Nextflow Report


- Summary
- Resources
- Tasks

[mad\_bell]

# Nextflow workflow report

## `[mad_bell]` *(resumed run)*

#### Workflow execution completed unsuccessfully!

The exit status of the task that caused the workflow execution to fail was: `1`.

The full error message was:

```
Error executing process > 'NFCORE_HIVGENOPIPE:HIVGENOPIPE:REPORT_STANFORD_JSON (Sample_8_T1)'

Caused by:
  Process `NFCORE_HIVGENOPIPE:HIVGENOPIPE:REPORT_STANFORD_JSON (Sample_8_T1)` terminated with an error exit status (1)

Command executed:

  stanford_json.py -f Sample_8_T1.Amb15.fasta Sample_8_T1.Amb2.fasta Sample_8_T1.Amb5.fasta Sample_8_T1.consensus.fasta

Command exit status:
  1

Command output:
  Getting characterization data from Stanford for Sample_8_T1.Amb15

Command error:
      return _default_decoder.decode(s)
             ^^^^^^^^^^^^^^^^^^^^^^^^^^
    File "/usr/local/lib/python3.11/json/decoder.py", line 337, in decode
      obj, end = self.raw_decode(s, idx=_w(s, 0).end())
                 ^^^^^^^^^^^^^^^^^^^^^^^^^^^^^^^^^^^^^^
    File "/usr/local/lib/python3.11/json/decoder.py", line 355, in raw_decode
      raise JSONDecodeError("Expecting value", s, err.value) from None
  json.decoder.JSONDecodeError: Expecting value: line 1 column 1 (char 0)
  
  During handling of the above exception, another exception occurred:
  
  Traceback (most recent call last):
    File "/usr/local/lib/python3.11/site-packages/gql/transport/requests.py", line 243, in execute
      result = response.json()
               ^^^^^^^^^^^^^^^
    File "/usr/local/lib/python3.11/site-packages/requests/models.py", line 975, in json
      raise RequestsJSONDecodeError(e.msg, e.doc, e.pos)
  requests.exceptions.JSONDecodeError: Expecting value: line 1 column 1 (char 0)
  
  During handling of the above exception, another exception occurred:
  
  Traceback (most recent call last):
    File "/mnt/LHRI/Bioinformatics/Projects/HIVGenoPipe/github/HIVGenoPipe_Pub/bin/stanford_json.py", line 76, in 
      main()
    File "/mnt/LHRI/Bioinformatics/Projects/HIVGenoPipe/github/HIVGenoPipe_Pub/bin/stanford_json.py", line 70, in main
      get_seq(args.fastas)
    File "/mnt/LHRI/Bioinformatics/Projects/HIVGenoPipe/github/HIVGenoPipe_Pub/bin/stanford_json.py", line 42, in get_seq
      data = client.execute(gql(open(os.path.join(dir_path,'sierra.gql')).read()),variable_values={"sequences": [currSeq]})
             ^^^^^^^^^^^^^^^^^^^^^^^^^^^^^^^^^^^^^^^^^^^^^^^^^^^^^^^^^^^^^^^^^^^^^^^^^^^^^^^^^^^^^^^^^^^^^^^^^^^^^^^^^^^^^^
    File "/usr/local/lib/python3.11/site-packages/sierrapy/sierraclient.py", line 69, in execute
      result: Dict[str, Any] = self.client.execute(
                               ^^^^^^^^^^^^^^^^^^^^
    File "/usr/local/lib/python3.11/site-packages/gql/client.py", line 403, in execute
      return self.execute_sync(
             ^^^^^^^^^^^^^^^^^^
    File "/usr/local/lib/python3.11/site-packages/gql/client.py", line 220, in execute_sync
      with self as session:
    File "/usr/local/lib/python3.11/site-packages/gql/client.py", line 702, in __enter__
      return self.connect_sync()
             ^^^^^^^^^^^^^^^^^^^
    File "/usr/local/lib/python3.11/site-packages/gql/client.py", line 686, in connect_sync
      self.session.fetch_schema()
    File "/usr/local/lib/python3.11/site-packages/gql/client.py", line 881, in fetch_schema
      execution_result = self.transport.execute(parse(get_introspection_query()))
                         ^^^^^^^^^^^^^^^^^^^^^^^^^^^^^^^^^^^^^^^^^^^^^^^^^^^^^^^^
    File "/usr/local/lib/python3.11/site-packages/gql/transport/requests.py", line 249, in execute
      raise_response_error(response, "Not a JSON answer")
    File "/usr/local/lib/python3.11/site-packages/gql/transport/requests.py", line 236, in raise_response_error
      raise TransportProtocolError(
  gql.transport.exceptions.TransportProtocolError: Server did not return a GraphQL result: Not a JSON answer: 

Work dir:
  /array/Members/dotrangtl/082124_LD_30_Sanger_test/work/d1/9eb30d4ef852b2ccb537aaa74a890e

Tip: you can try to figure out what's wrong by changing to the process work dir and showing the script file named `.command.sh`
```

Run times
:   15-May-2025 15:42:19 - 15-May-2025 16:32:51
    (duration: **50m 32s**)

693 succeeded

163 cached

1 ignored

2 failed

Nextflow command
:   ```
    nextflow run /mnt/LHRI/Bioinformatics/Projects/HIVGenoPipe/github/HIVGenoPipe_Pub/main.nf --input LD_30_Sanger_samplesheet_clean_controls.csv --fasta ../test_data/resources/K03455.1_HXB2_737-5219.fasta --positive_control_ref ../test_data/resources/NL4_3.fasta -profile docker --outdir ./05092025_30_samples_amb2_run -resume
    ```

CPU-Hours
:   `16.3 (28.7% cached, 0.2% failed)`

Launch directory
:   `/array/Members/dotrangtl/082124_LD_30_Sanger_test`

Work directory
:   `/array/Members/dotrangtl/082124_LD_30_Sanger_test/work`

Project directory
:   `/mnt/LHRI/Bioinformatics/Projects/HIVGenoPipe/github/HIVGenoPipe_Pub`

Script name
:   `main.nf`

Script ID
:   `6c46fa9cd08761b5de27f8fcbed649c4`

Workflow session
:   `2a76e6f1-25a2-4b99-a2fa-51ccc2da19d2`

Workflow profile
:   docker

Nextflow version
:   version 23.10.1, build 5891 (12-01-2024 22:01 UTC)

## Resource Usage

These plots give an overview of the distribution of resource usage for each process.

#### CPU

- Raw Usage
- % Allocated

#### Memory

- Physical (RAM)
- Virtual (RAM + Disk swap)
- % RAM Allocated

#### Job Duration

- Raw Usage
- % Allocated

#### I/O

- Read
- Write

## Tasks

This table shows information about each task in the workflow. Use the search box on the right
to filter rows for specific values. Clicking headers will sort the table by that value and
scrolling side to side will reveal more columns.

Values shown as:

Human readable
Raw values

(tasks table omitted because the dataset is too big)

Generated by Nextflow, version 23.10.1
