# Supplementary material for: HIVGenoPipe: a nextflow pipeline for the detection of HIV-1 drug resistance using a real-time sample-specific reference sequence
Source: BMC Bioinformatics. 2025 Jul 7;26:168. doi: 10.1186/s12859-025-06201-5 (PMC12235847; doi:10.1186/s12859-025-06201-5)
Supplement: Supplementary file 1 — Additional file 1. [file 12859_2025_6201_MOESM1_ESM.zip › Supplemental Data 2-Example_Output.zip!/Supplemental_Data_Example_Output/pipeline_info/pipeline_dag_2025-05-09_13-32-47.html]

```
flowchart TB
    subgraph " "
    v2["Channel.fromPath"]
    v7[" "]
    v10["samplesheet"]
    v34[" "]
    v40["sort_bam"]
    v93["sort_bam"]
    v102["sort_bam"]
    v140["sort_bam"]
    v176["Channel.from"]
    v181["ch_workflow_summary"]
    end
    subgraph " "
    v3["ch_positive_control_ref_fasta"]
    v12["versions"]
    v16[" "]
    v20[" "]
    v21[" "]
    v25[" "]
    v26[" "]
    v27[" "]
    v28[" "]
    v30[" "]
    v33[" "]
    v45[" "]
    v46[" "]
    v49["versions"]
    v56[" "]
    v57[" "]
    v59[" "]
    v61[" "]
    v70[" "]
    v76[" "]
    v81[" "]
    v82[" "]
    v83[" "]
    v85[" "]
    v89[" "]
    v91[" "]
    v98[" "]
    v100[" "]
    v104[" "]
    v106[" "]
    v107[" "]
    v108[" "]
    v113[" "]
    v116[" "]
    v120[" "]
    v122[" "]
    v124[" "]
    v137[" "]
    v139[" "]
    v142[" "]
    v144[" "]
    v146[" "]
    v151[" "]
    v165[" "]
    v167["db_samtools_stats"]
    v169["db_jsons"]
    v173[" "]
    v174[" "]
    v194[" "]
    v195[" "]
    v197[" "]
    v199["ch_versions"]
    end
    subgraph NFCORE_HIVGENOPIPE
    subgraph HIVGENOPIPE
    subgraph INPUT_CHECK
    v11([SAMPLESHEET_CHECK])
    v13(( ))
    end
    v15([FASTQC])
    v19([TRIMMOMATIC])
    v24([BBTOOLS_SEAL])
    subgraph CONTROL_TEST
    v37([BWA_INDEX])
    v41([BWA_MEM])
    v44([SAMTOOLS_INDEX])
    v54([PYSAMSTATS])
    v55([PYSAMSTATS_PARSER])
    v58([SAMTOOLS_STATS])
    v60([SAMTOOLS_DEPTH])
    v62([REPORT_CHECK_COVERAGE])
    v66([REPORT_SAMTOOLS_DEPTH_AND_STATS])
    v69([REPORT_NGS_STATS])
    v31(( ))
    v35(( ))
    v38(( ))
    v52(( ))
    v65(( ))
    end
    v75([REPORT_NGS_STATS_ALL])
    v77([TRINITY])
    v80([BBMAP_BBDUK])
    v84([MAFFT])
    v88([MAFFT_CONSENSUS])
    v90([BWA_INDEX_1])
    v94([BWA_MEM_1])
    v97([SAMTOOLS_CONSENSUS])
    v99([BWA_INDEX_2])
    v103([BWA_MEM_2])
    v105([SAMTOOLS_INDEX])
    v112([SAMTOOLS_STATS])
    v114([PYSAMSTATS])
    v115([PYSAMSTATS_PARSER])
    v117([REPORT_CHECK_COVERAGE])
    v118([REPORT_STANFORD_JSON])
    v119([REPORT_STANFORD_SUMMARY])
    v121([SAMTOOLS_DEPTH_1])
    v123([REPORT_PLOT_DEPTH_1])
    v128([REPORT_SAMTOOLS_DEPTH_AND_STATS])
    v136([REPORT_NGS_STATS_PASS])
    v138([BWA_INDEX_REF])
    v141([BWA_MEM_REF])
    v143([SAMTOOLS_DEPTH_REF])
    v145([REPORT_PLOT_DEPTH_REF])
    v149([REPORT_SIMILARITY])
    v150([REPORT_SIMILARITY_MATRIX])
    v164([REPORT_SUMMARY])
    v172([CUSTOM_DUMPSOFTWAREVERSIONS])
    v193([MULTIQC])
    v8(( ))
    v17(( ))
    v29(( ))
    v71(( ))
    v74(( ))
    v92(( ))
    v101(( ))
    v109(( ))
    v127(( ))
    v131(( ))
    v147(( ))
    v166(( ))
    v168(( ))
    v177(( ))
    v196(( ))
    end
    end
    v2 --> v3
    v7 --> v8
    v10 --> v11
    v11 --> v12
    v11 --> v13
    v13 --> v15
    v15 --> v16
    v15 --> v17
    v15 --> v177
    v13 --> v19
    v19 --> v24
    v19 --> v21
    v19 --> v20
    v19 --> v77
    v19 --> v141
    v19 --> v17
    v19 --> v29
    v19 --> v31
    v19 --> v74
    v19 --> v92
    v19 --> v101
    v19 --> v131
    v8 --> v24
    v24 --> v28
    v24 --> v27
    v24 --> v26
    v24 --> v25
    v24 --> v31
    v24 --> v74
    v24 --> v131
    v29 --> v30
    v31 --> v33
    v34 --> v35
    v35 --> v37
    v37 --> v41
    v37 --> v38
    v40 --> v41
    v29 --> v41
    v41 --> v44
    v41 --> v60
    v41 --> v38
    v41 --> v52
    v44 --> v46
    v44 --> v45
    v44 --> v38
    v44 --> v52
    v38 --> v49
    v35 --> v54
    v52 --> v54
    v54 --> v55
    v55 --> v57
    v55 --> v56
    v55 --> v62
    v52 --> v58
    v58 --> v59
    v58 --> v65
    v60 --> v61
    v60 --> v65
    v35 --> v62
    v62 --> v31
    v65 --> v66
    v66 --> v31
    v31 --> v69
    v69 --> v70
    v69 --> v71
    v74 --> v75
    v75 --> v76
    v75 --> v71
    v77 --> v80
    v77 --> v17
    v8 --> v80
    v80 --> v84
    v80 --> v83
    v80 --> v82
    v80 --> v81
    v8 --> v84
    v84 --> v88
    v84 --> v85
    v84 --> v17
    v88 --> v90
    v88 --> v89
    v90 --> v91
    v90 --> v92
    v93 --> v94
    v92 --> v94
    v94 --> v97
    v94 --> v17
    v97 --> v99
    v97 --> v98
    v97 --> v109
    v99 --> v100
    v99 --> v101
    v102 --> v103
    v101 --> v103
    v103 --> v105
    v103 --> v104
    v103 --> v121
    v103 --> v109
    v103 --> v131
    v105 --> v108
    v105 --> v107
    v105 --> v106
    v105 --> v109
    v109 --> v112
    v112 --> v113
    v112 --> v127
    v112 --> v166
    v112 --> v177
    v109 --> v114
    v114 --> v115
    v115 --> v116
    v115 --> v118
    v115 --> v117
    v115 --> v131
    v115 --> v147
    v8 --> v117
    v117 --> v131
    v118 --> v119
    v118 --> v168
    v119 --> v120
    v121 --> v123
    v121 --> v122
    v121 --> v127
    v123 --> v124
    v127 --> v128
    v128 --> v131
    v131 --> v136
    v136 --> v137
    v136 --> v71
    v8 --> v138
    v138 --> v141
    v138 --> v139
    v140 --> v141
    v141 --> v143
    v141 --> v142
    v143 --> v145
    v143 --> v144
    v145 --> v146
    v147 --> v149
    v149 --> v150
    v150 --> v151
    v71 --> v164
    v164 --> v165
    v166 --> v167
    v168 --> v169
    v17 --> v172
    v172 --> v174
    v172 --> v173
    v172 --> v177
    v176 --> v177
    v181 --> v177
    v177 --> v193
    v193 --> v195
    v193 --> v194
    v193 --> v17
    v193 --> v196
    v196 --> v197
    v17 --> v199
```
